# Supramolecular Rings as Building Blocks for Stimuli-Responsive Materials

**DOI:** 10.3390/gels8060350

**Published:** 2022-06-03

**Authors:** Hanna Traeger, Alyssa Ghielmetti, Yoshimitsu Sagara, Stephen Schrettl, Christoph Weder

**Affiliations:** 1 Adolphe Merkle Institute, University of Fribourg, Chemin des Verdiers 4, 1700 Fribourg, Switzerland; hanna.traeger@unifr.ch (H.T.); alyssa.ghielmetti@ugent.be (A.G.); 2 Department of Materials Science and Engineering, Tokyo Institute of Technology, 2-12-1 Ookayama, Meguro-ku, Tokyo 152-8552, Japan; sagara.y.aa@m.titech.ac.jp

**Keywords:** stimuli-responsive polymers, chromogenic, fluorescence, luminescence, quenching, benzothiadiazole, naphthalene diimide

## Abstract

Stimuli-responsive polymers are of great interest due to their ability to translate changing environmental conditions into responses in defined materials. One possibility to impart such behavior is the incorporation of optically active molecules into a polymer host. Here, we describe how sensor molecules that consist of a π-extended benzothiadiazole emitter and a naphthalene diimide quencher can be exploited in this context. The two optically active entities were connected via different spacers and, thanks to attractive intramolecular interactions between them, the new sensor molecules assembled into cyclic structures in which the fluorescence was quenched by up to 43% when compared to solutions of the individual dyes. Detailed spectroscopic investigations of the sensor molecules in solution show that the extent of donor/acceptor interactions is influenced by various factors, including solvent polarity and ion concentration. The new sensor molecule was covalently incorporated into a polyurethane; the investigation of the optical characteristics in both the solid and solvent-swollen states indicates that a stimulus-induced formation of associated dye pairs is possible in polymeric materials. Indeed, a solvatochromic quenching effect similar to the behavior in solution was observed for solvent-swollen polymer samples, leading to an effective change of the green emission color of the dye to a yellow color.

## 1. Introduction

Chromogenic polymers can change their visible color, fluorescence color, or emission intensity in response to external stimuli [1]. Such materials are of great interest as sensors that translate an applied stimulus into a defined optical response. One general approach toward chromogenic polymers is the incorporation of chromophores that change their optical properties upon interaction with another chromophore of the same or different type [2,3,4,5,6]. Indeed, temperature changes, light irradiation, mechanical force, or the changing polarity of the environment can serve as stimuli that lead to the assembly or disassembly of the chromophores, which can in turn trigger a defined optical response in the material. Chromophore systems that have been used in this context include excimer-forming dyes [7,8], energy-transfer pairs [9], charge-transfer complexes [10], and photo-induced electron transfer dye pairs [11]. The corresponding polymers featuring these dye systems have been used to sense, e.g., temperature [12], chemicals [13,14], and mechanical deformation [8,9,11,15,16,17,18]. Whereas simple blending of polymers with a suitable dye is often sufficient to create responsive materials [2,3,4,7], it can be beneficial to covalently incorporate the chromophores into a polymer matrix. This allows, for example, the prevention of phase separation and aggregation of the dyes, and can be helpful in ensuring an efficient translation of mechanical stimuli into an optical response [19,20,21,22,23,24]. Indeed, the interacting chromophores can be more directly affected when efficient transduction of macroscopically-applied forces to the molecular-level sensor is ensured. This has, for example, been demonstrated recently with several rotaxane-based displacement mechanophores [11,15,16,17], as well as with loop-forming structures containing two emitters capable of excimer formation [25,26]. The investigated rotaxane-based motifs combine a macrocycle equipped with a fluorophore and a dumbbell-shaped axle with a suitable quencher. Such rotaxanes do not fluoresce in the resting state because the fluorophore is in close proximity to the quencher; thus, emission is suppressed through the formation of charge-transfer complexes and/or a photoinduced electron transfer process. Elastic polymers containing such rotaxanes display an instant and reversible fluorescence turn-on upon deformation as a result of the spatial separation of the interacting dye pair upon deformation. Unfortunately, the complex synthesis of such rotaxanes impedes their widespread use. In contrast, loop-forming mechanophores based on perylene dyes can be straightforwardly prepared in as little as three synthetic steps [25,26]. The loop-forming motif was incorporated into polyacrylates and different polyurethanes, which imparts the materials with mechanochromic properties. In the relaxed state the red excimer emission of the associated dye pairs is observed, while the fluorescence color changes to the green monomer-centered emission upon mechanical deformation. Spectroscopic analyses, moreover, show that polymers featuring the mechanophore reliably translate the applied strain into a defined optical signal.

**Figure 1:**
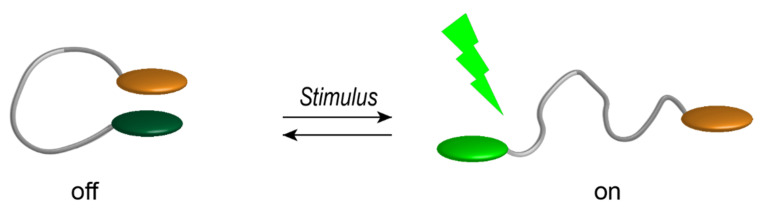
Schematic representation of a covalently connected dye pair that may form an intramolecular cyclic structure as a sensor element. In the idle state, the two dyes are in proximity and the fluorescence emission is quenched. Upon exposure to a suitable stimulus, the fluorophore and quencher are separated and the emission of the former is switched.

In an alternative approach to the use of perylene dyes, we surmised that the chromogenic response enabled by the weak, non-covalent association of the fluorophore and quencher pairs employed in the previously-reported rotaxanes could be exploited in loop-type structures that are much easier to access. Accordingly, we set out to explore new types of linear molecules in which dye pairs are covalently connected by a short linker (Figure 1). The intramolecular association of the chromophores, which can be influenced via the interaction strength of the two motifs [7,21] as well as by auxiliary groups that provide directional non-covalent interactions [9,18], might lead to the formation of dynamic cyclic structures with quenched or altered fluorescence emission. Such motifs could display straightforward stimulus-induced assembly or disassembly, and appear to be attractive candidates for the development of a wide range of novel stimuli-responsive sensor motifs, notably, mechanochromic mechanophores [8,9,10,18,25,26,27].

## 2. Results and Discussion

A π-extended benzothiadiazole (emitter) and a naphthalene diimide (quencher) were selected to test the concept of supramolecular rings as responsive motif, because the combination of these dyes in a rotaxane has recently been demonstrated to afford a mechanophore with interesting fluorescence switch-on capabilities [11]. Molecular modelling and simple universal force field (UFF) energy optimization suggests that both a hydrocarbon linker of C_12_ or longer and ethylene oxide linkers with six or more repeating units are suitable to allow for an unconstrained interaction between the two chromophores (Figure S1). The molecules must be designed to feature either peripheral double bonds or hydroxy groups to allow for incorporation into polymers via thiol-ene click chemistry or their use as co-monomers in the preparation of polyurethanes.

**Scheme 1:**
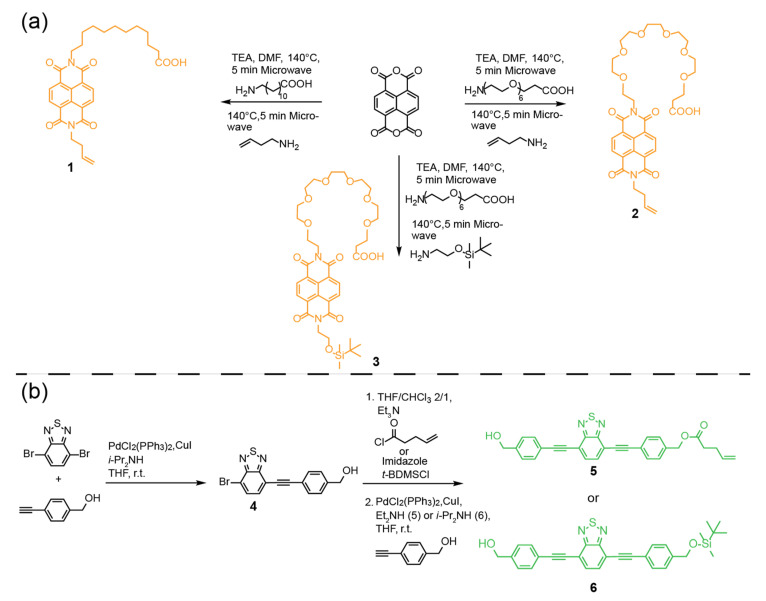
(**a**) Synthesis of the naphthalene diimide building blocks **1**, **2**, and **3** from naphthalenetetracarboxylic dianhydride via condensation with different amines in microwave-assisted reactions. (**b**) Synthesis of the asymmetrically functionalized benzothiadiazole emitter derivatives **5** and **6** via Sonogashira coupling.

**Scheme 2:**
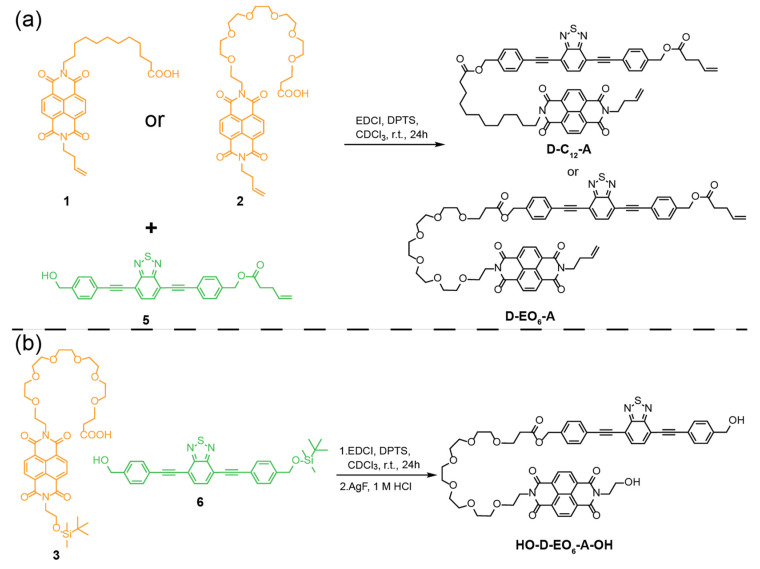
(**a**) Synthesis of the new sensor molecules featuring a benzothiadiazole emitter and a naphthalene diimide quencher connected by a hydrocarbon linker (**D-C_12_-A**) or an oligo(ethylene oxide) linker (**D-EO_6_-A**). (**b**) Synthesis of a hydroxy-functionalized fluorophore–quencher pair (**HO-D-EO_6_-A-OH**).

The synthetic pathway by which these new sensor molecules were accessed is shown in Scheme 1 and Scheme 2. Microwave-assisted syntheses were carried out in order to access three different asymmetrically-substituted naphthalene diimide derivatives (**1**–**3**) in one-step reactions between naphthalenetetracarboxylic dianhydride and different amines [28,29]. In order to synthesize the naphthalene diimide derivative **1**, substituted with a terminal double bond and a C_12_-spacer with a terminal carboxylic acid, the reaction was carried out with a combination of 3-buten-1-amine and 12-aminododecanoic acid (Scheme 1a).

Alternatively, amino-EO_6_-carboxylic acid was used in combination with 3-buten-1-amine to obtain access to **2** and with *tert*-butyldimethylsilyl (*t-*BDMS) ether of ethanolamine to furnish **3**. The π-extended benzothiadiazole **4** was prepared by Sonogashira coupling of 4,7-dibromo-2,1,3-benzothiadiazole and 4-ethynylbenzyl alcohol [30,31], and subjected to esterification with pent-4-enoyl chloride and a second Sonogashira coupling with 4-ethynylbenzyl alcohol to afford the emitter **5** (Scheme 1b). Moreover, a second benzothiadiazole derivative, **6**, was prepared via protection of the hydroxy functional group of **4** as a *t-*BDMS ether.

**Figure 2:**
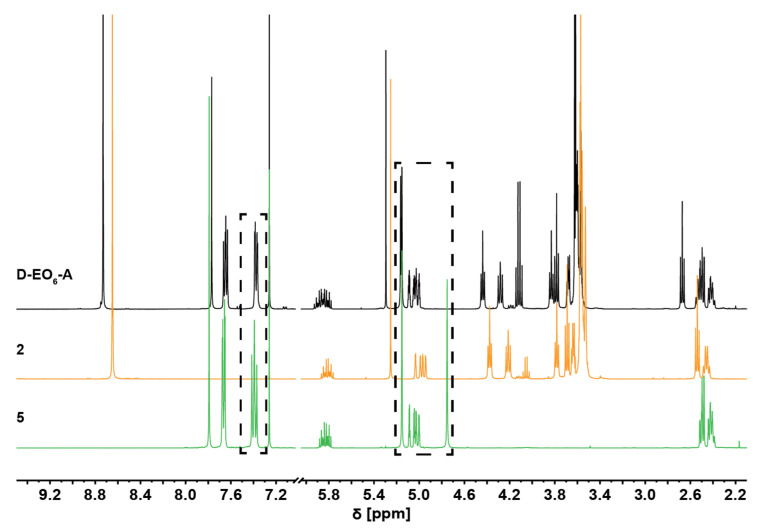
Comparison of the ^1^H NMR spectra of **D-EO_6_-A** (black line), **2** (orange line), and **5** (green line). The comparison of the signals highlighted by the boxes indicates the successful formation of the covalently connected fluorophore–quencher pair. The splitting pattern of the aromatic region of **5** (around 7.3 ppm) and the down-field shift of the signals associated with the –CH_2_-OH methylene group after esterification (around 5 ppm) are diagnostic for the successful formation of **D-EO_6_-A**.

Esterification reactions between the various alcohol and acid-terminated building blocks were carried out to prepare the target molecules, in which the emitter and quencher dye motifs are linked by a spacer. Benzothiadiazoles are known as electronically deficient emitters [30,31], which act as donors (**D**) with respect to the quenching naphthalene diimde acceptor (**A**) in the present system. A carbodiimide-mediated reaction between the naphthalene diimides **1** or **2** and the benzothiadiazole **5** was carried out to access **D-C_12_-A** and **D-EO_6_-A**, which feature an aliphatic and an oligo(ethylene oxide) linker, respectively (Scheme 2a). The successful formation of the target compounds was confirmed by ^1^H and ^13^C NMR spectroscopy and mass spectrometry (Figure 2 and Figure S2). Compared to the ^1^H NMR spectrum of **5**, the splitting pattern of the aromatic protons at ca. 7.3 ppm changes from a triplet to a doublet and the R–C*H*_2_–OH methylene signal undergoes an up-field shift after esterification with **1** or **2** from around 5 ppm (Figure 2). The carbodiimide-mediated esterification of **3** and **6** was followed by cleavage of the *t*-BDMS protecting groups via reaction with silver fluoride and hydrochloric acid, providing access to the hydroxy-terminated derivative **HO-D-OE_6_-A-OH** (Scheme 2b). The successful preparation of **HO-D-OE_6_-A-OH** was confirmed by NMR spectroscopy (Figure S3).

**Figure 3:**
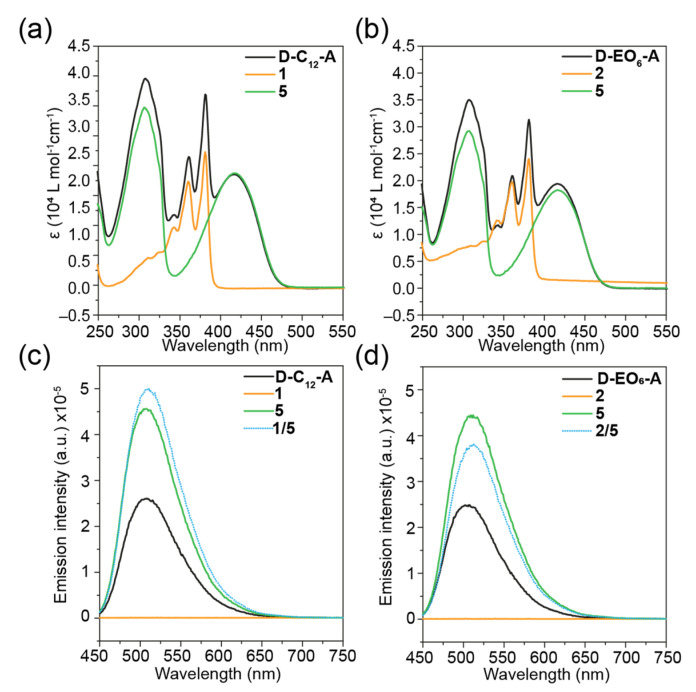
(**a**,**b**) Comparison of the UV-vis absorption spectra of (**a**) **D-C_12_-A**, **1**, and **5** and (**b**) of **D-EO_6_-A**, **2**, and **5**. (**c**,**d**) Comparison of the fluorescence spectra of (**c**) **D-C_12_-A**, **1**, **5**, and an equimolar mixture of **1** and **5** and (d) of **D-EO_6_-A**, **2**, **5**, and an equimolar mixture of **2** and **5**. All spectra were recorded in a mixture of CHCl_3_ and MeOH (99.5:0.5 *v*/*v*%) and the concentration of all dyes was 10 µmol L^−1^. All emission spectra were recorded with excitation at a wavelength (*λ*_ex_) of 417 nm.

In order to explore the association of the π-extended benzothiadiazole (emitter) and the naphthalene diimide (quencher), dilute solutions of **D-C_12_-A** and **D-EO_6_-A** in a mixture of CHCl_3_ and MeOH (99.5:0.5 *v*/*v*%) were characterized by UV-vis absorption and fluorescence spectroscopy and the data compared with the corresponding spectra of the naphthalene diimides **1** or **2**, the π-extended benzothiadiazole **5**, and equimolar mixtures of **1** or **2** and **5** (the concentration of all compounds was adjusted to *c* = 10 µmol L^−1^). A comparison of the UV-vis absorption spectra of the solutions shows that the spectra of **D-C_12_-A** and **D-EO_6_-A** mirror each other (Figure 3a,b). Moreover, the absorption spectra of the covalently connected quencher–emitter pairs appear to be superpositions of the spectra of the individual dye motifs **1** or **2** and **5** (Figure 3a,b). In order to record the fluorescence spectra of these solutions, excitation wavelengths (*λ*_ex_) of 417, 405, 400, and 385 nm were screened. Based on the recorded spectra for the different solutions (Figures S4 and S5), all emission spectra were subsequently recorded with an excitation wavelength of 417 nm. At this wavelength, only the emitter moieties absorb light and are electronically excited, which enables direct assessment of any quenching effects by comparing the emission intensities of the various solutions. The recorded spectra show that the emission intensity at 505 nm shows only minor changes when quenchers **1** or **2** are added to emitter **5**, i.e., in solutions containing separated emitter–quencher pairs, whereas a considerable reduction of the emission intensity to ca. 43% of that of **5** is seen in **D-C_12_-A** and **D-EO_6_-A** (Figure 3c,d). We note that the quenching effect is less pronounced than in the corresponding rotaxanes comprising the same quencher–emitter pair, in which the quenching effect is almost quantitative [11].

To exclude the possibility that the observed quenching effect was caused by intermolecular interactions, aliquots of either **1** or **2** were added to solutions of **5** and the emission intensity was monitored (Figure S6). No reduction in fluorescence intensity was observed, even after addition of an excess of 40 equivalents of the different naphthalene diimide derivatives to solutions of **5**. Moreover, further reference experiments showed that the reduced emission intensity of solutions of **D-C_12_-A** and **D-EO_6_-A** is independent of changes in temperature (Figures S7–S9) and concentration (Figure S10) within the experimentally explored conditions. Thus, the data show unequivocally that the quenching of the emission of the benzothiadiazole moiety in the investigated solutions of **D-C_12_-A** and **D-EO_6_-A** is due to an intramolecular effect that originates from the close proximity of the emissive benzothiadiazole and the quenching naphthalene diimide, which indicates the formation of dynamic cycles (Figure 1). This interpretation appears to be further supported by the observed shift in the positions of the ^1^H NMR signals of **D-EO_6_-A** compared to the signals observed for the quencher **2** (Figure 2).

**Figure 4:**
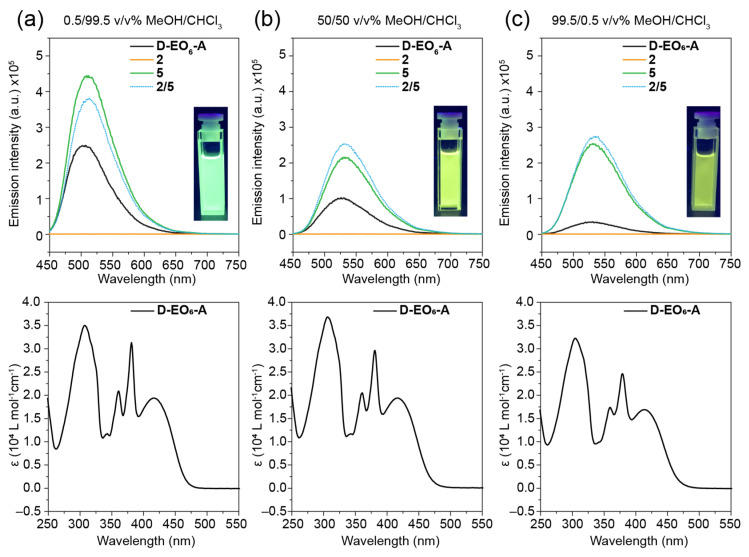
Emission spectra (**top**) and UV-vis absorption spectra (**bottom**) of **D-EO_6_-A** and reference compounds **2** and **5** as well as of equimolar mixtures of **2/5** in MeOH/CHCl_3_ solutions of (**a**) 0.5:99.5, (**b**) 50:50, and (**c**) 99.5:0.5 *v*/*v*%. The insets show photographs of the solutions that were illuminated by UV light (365 nm). The concentration of all solutions was 10 µmol L^−1^, and an excitation wavelength (*λ*_ex_) of 417 nm was used.

With these findings in hand, the influence of the solvent polarity and the effect of inorganic salts on the emission characteristics of **D-C_12_-A** and **D-EO_6_-A** were investigated. Solutions of **D-C_12_-A**, the individual compounds **1** and **5**, and the mixture **1/5** in different mixtures of methanol and chloroform were thus investigated; the MeOH/CHCl_3_ ratio was varied from 0.5:99.5 to 50:50 to 99.5:0.5 *v*/*v*% (Figure S11). Compared to solutions of **5** and the mixture **1/5**, spectra of solutions of **D-C_12_-A** display a pronounced decrease in the fluorescence intensity of the band at 505 nm and a concomitant bathochromic shift to 530 nm with increasing solvent polarity (Figure S11). These changes are reflected in photographs of the cuvettes taken under UV-light illumination (*λ*_ex_ = 365 nm, Figure S11). In solutions with a mixture of 99.5:0.5 *v*/*v*% MeOH/CHCl_3_ the emission is completely quenched; however, the changes observed in the absorption spectra in the various solvent mixtures suggest that the reason for this behavior is an aggregation of **D-C_12_-A** on account of decreased solubility in the polar solvent mixture (Figure S11). Notably, however, the recorded fluorescence spectra of solutions of **D-EO_6_-A**, which exhibits an increased solubility in polar solvents, suggest similarly pronounced emission quenching with increasing solvent polarity (Figure 4). Indeed, the corresponding UV-vis absorption spectra in solutions of varying polarity mirror each other, suggesting that **D-EO_6_-A** remains molecularly dissolved in these solutions. Thus, at least in solutions of **D-EO_6_-A** in polar solvent mixtures, the attractive intramolecular interactions among the benzothiadiazole and naphthalene diimide motifs lead to the formation of supramolecular cycles, in which the emission of the benzothiadiazole emitters is efficiently quenched.

**Figure 5:**
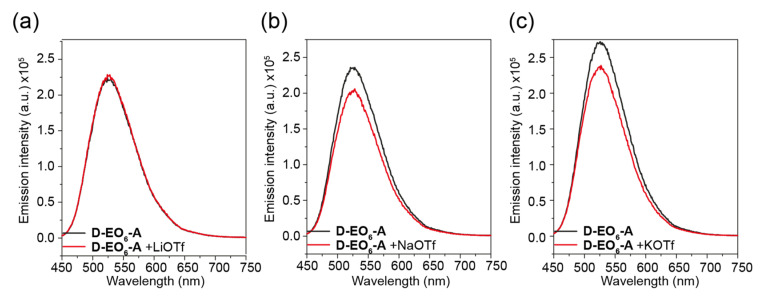
Emission spectra (*λ*_ex_ = 417 nm) of solutions of **D-EO_6_-A** (*c* = 10 µmol L^−1^, in MeOH/CHCl_3_ 50:50 *v*/*v*%) before (black lines) and after (red lines) addition of 5 mg of the metal salts (**a**) LiOTf, (**b**) NaOTf, and (**c**) KOTf.

Since oligo(ethylene oxide)s are known chelators of cationic alkaline or alkaline earth metal ions [32], effects caused by the addition of the triflate salts of lithium, sodium, potassium, barium, magnesium, and calcium on the fluorescence emission of **D-EO_6_-A** were explored. The same experiments were carried out for reference purposes with **D-C_12_-A**, as its short alkyl spacer between the quencher-emitter pair should not promote binding to these metal ions. Emission spectra were recorded in MeOH/CHCl_3_ (50:50 *v*/*v*%) solutions of **D-EO_6_-A** (Figure 5 and Figure S12) and **D-C_12_-A** (Figure S13) before and after addition of 5 mg of the different metal salts.

This solvent mixture was selected because the formation of cyclic structures is (vide supra) incomplete in the absence of any metal salt. As expected, the addition of the various metal salts had no impact on the emission intensity of **D-C_12_-A** (Figure S12). In contrast, the benzothiadiazole emission was slightly reduced upon addition of sodium and potassium salts to solutions of **D-EO_6_-A** (Figure 5), which is consistent with the coordination of the cations by the oligo(ethylene oxide) linker and a concomitantly increased proximity between the donor and acceptor moieties [33], as previously reported for charge-transfer complexes [34,35].

**Scheme 3:**
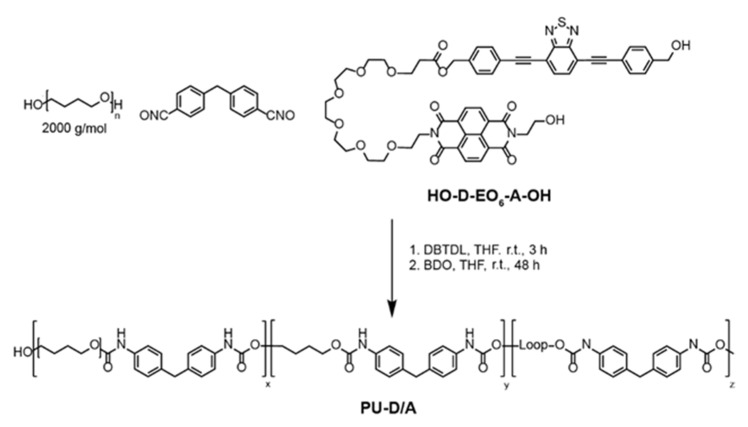
Synthesis of a polyurethane containing the loop-forming sensor moiety. In a pre-polymerization step, **HO-D-EO_6_-A-OH** and 2000 g mol^−1^ poly(tetrahydrofuran) (p(THF)) were reacted, with an excess of methylene diphenyl diisocyanate (MDI) using dibutyltin laureate (DBTL) as catalyst. After 3 h, 1,4-butanediol (BDO) was added as a chain extender and the polymerization reaction was allowed to continue for 48 h at room temperature, yielding **PU-D/A**.

To incorporate the new motifs into polymers in which their self-assembly would lead to the formation of intramolecular loops, we initially explored the suitability of the terminal double bonds in **D-C_12_-A** and **D-EO_6_-A** for thiol-ene click reactions with thiol-terminated PLA, however, this approach was unsuccessful (Figures S14–S15). No conversion of the double bonds could be observed, as indicated by a comparison of the ^1^H NMR spectra of **D-C_12_-A** recorded before and after the reaction (Figures S14 and S15). To overcome this issue, the incorporation of the hydroxy-terminated **HO-D-EO_6_-A-OH** into polyurethanes (PUs) was pursued (Scheme 3). Thus, a small amount (7 mg, 0.18 wt%) of **HO-D-EO_6_-A-OH** was employed as a co-monomer in the polyaddition reaction of poly(tetrahydrofuran) (p(THF)), methylene diphenyl diisocyanate (MDI), and butanediol (BDO; Scheme 3) [11,20]. The resulting polymer (**PU-D/A**) was characterized via size exclusion chromatography (SEC), ^1^H NMR spectroscopy, thermogravimetric analysis (TGA), and differential scanning calorimetry (DSC; Figure S16). SEC traces indicate a number-average molecular weight of *M*_n_ = 46 kg mol^−1^ and a dispersity of *Ð* = 1.9. The ^1^H NMR spectrum shows the characteristic PU signals, although due to the low concentration no signals of the **HO-D-EO_6_-A-OH** residues were observed. The recorded TGA traces indicate an onset of degradation at ca. 240 °C. The first DSC heating trace of samples of the as-prepared polymer, moreover, feature an endothermic transition at ca. 8 °C that is absent in all subsequent heating traces; the latter can be tentatively assigned to the melting transition of the semicrystalline p(THF) domains that form in solvent-cast samples.

**Figure 6:**
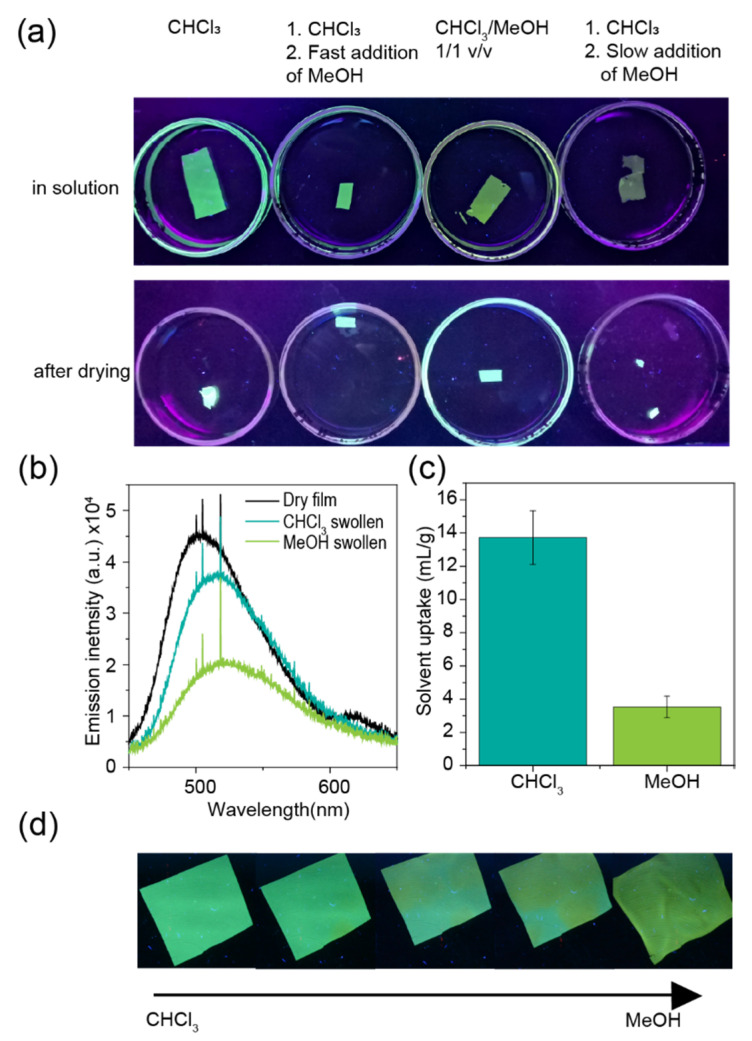
(**a**) Swelling experiments with samples of **PU-D/A** films. The top row shows images of solvent-cast films that were swollen by addition of (from left to right) CHCl_3_, CHCl_3_ with an excess of MeOH, an MeOH/CHCl_3_ (50:50 *v*/*v*%) mixture, and CHCl_3_ followed by the slow addition of MeOH. The bottom row shows the same samples after drying by solvent evaporation at room temperature. (**b**) Emission spectra (*λ*_ex_ = 305 nm) of a dry (black line), CHCl_3_-swollen (turquoise line), and MeOH-swollen (green line) film of **PU-D/A**. (**c**) MeOH and CHCl_3_ uptake of **PU-D/A** films determined gravimetrically after swelling of the samples for 5 h. Values represent averages of three individual measurements ± standard error of mean. (**d**) Photographs of a solvent-cast **PU-D/A** film that was swollen in CHCl_3_ (left) and images taken during dropwise addition of methanol. All images were recorded under UV light illumination (365 nm).

Thin films of **PU-D/A** were prepared by solvent casting from THF solutions. Similar PU-based materials that feature other dye systems have recently been reported, and the mechanical properties of such polymer films have been extensively described in several studies [8,11,15,16,17]. In order to determine whether the influence of solvent polarity on the self-assembly and emission characteristics observed in solution could be observed for **PU-D/A** films, solvent-cast films of the physically cross-linked PU were swollen in solvent mixtures of varying polarity, i.e., in CHCl_3_, CHCl_3_ (with fast addition of an excess of MeOH), MeOH/CHCl_3_ (50:50 *v*/*v*%), and CHCl_3_ (with slow addition of MeOH). Photographs of the different samples were taken under UV light illumination (365 nm) in both the swollen state of the physically cross-linked materials and after drying (Figure 6a).

The images show clearly that the solvatochromic effect is retained for **PU-D/A** films. Upon increasing the solvent polarity, the fluorescence intensity is reduced and the emission color is red-shifted (Figure 6b), as indicated by the green emission color of the CHCl_3_-swollen samples changing to yellow upon the slow addition of methanol. After drying, the light blue fluorescence of the solvent-cast films is restored, which indicates that the effect is neither driven by the extent of swelling (CHCl_3_ > MeOH > dry film) (Figure 6c) or by aggregation-induced quenching effects. To further demonstrate the solvatochromic change in the emission, a solvent-cast **PU-D/A** film was swollen in CHCl_3_ and the emission was monitored during the dropwise addition of methanol (Figure 6d). The still images from the recorded video show that the addition of methanol results in an immediate shift in fluorescence color with a concomitant decrease in emission intensity, corroborating the conclusion that increased polarity leads to an association of the donor and acceptor moieties in these physically cross-linked polymer films, presumably under the formation of intramolecular loops.

## 3. Conclusions

In summary, we report a facile method to synthetically access covalently connected dye pairs that combine acceptor and donor motifs into a linked structure along with an investigation into their spectroscopic properties. The two motifs, **D-C_12_-A** and **D-EO_6_-A**, combine a naphthalene diimide quencher with a benzothiadiazole emitter, and can be prepared in six steps from commercially available starting materials. Our detailed spectroscopic investigation shows that the covalent connection of emitting and quenching moieties via a spacer of appropriate length affords molecules than can self-assemble into (dynamic) cyclic structures via intramolecular association of the donor and acceptor moieties. The extent of ring formation, and thus of quenching of the benzothiadiazole emission, depends on solvent polarity and can be controlled by other factors that affect the association of the dyes. The new sensor molecules were incorporated into the backbone of a polyurethane, and a similar solvatochromic quenching effect was observed for swollen polymer films to which polar solvents were added. Based on these results as well as on very promising studies with structurally related covalently-linked dye pairs [10,25,26,27], we speculate that future variants of such sensor molecules with increased interactions among the quencher and emitter moieties might afford polymer systems in which the donor–acceptor pairs are fully assembled in the idle state; the resulting molecular loops should serve as efficient sensors for mechanical deformation.

## 4. Materials and Methods

### 4.1. Materials

Unless stated otherwise, all chemicals and solvents were purchased at a purity of 95% or higher and used without further purification. Reactions were carried out using dried Schlenk glassware under nitrogen atmosphere. Deuterated solvents were purchased from Cambridge Isotope Laboratories, Inc. or Sigma Aldrich. Naphthalene-1,4,5,8-tetracarboxylic dianhydride, 12-aminolauric acid, and 4,7-dibromo-2,1,3-benzothiadiazole were purchased from TCI. (Triethyl)amine, hydrochloric acid (conc.), copper (I) iodide, (diisopropyl)amine, 4-ethynylbenzyl alcohol, dry (diethyl)amine, dry (triethyl)amine, 4-pentenoyl chloride, imidazole, *tert*-butyldimethylsilyl chloride, 1-ethyl-3-(3-dimethylaminopropyl)carbodiimid hydrochloride, poly(tetrahydrofuran), 4,4′-methylenebis(phenylisocyanate), dibutyltin dilaurate, 1,4-butanediol, and the cross-coupling catalyst PdCl_2_(PPh_3_)_2,_ 2-((*tert*-butyldimethylsilyl)oxy)ethanamine were purchased from Sigma Aldrich. The solvents *N,N′*-dimethylformamide, acetone, methanol, tetrahydrofuran, chloroform, and ethyl acetate for reactions and for solution-phase spectroscopy were purchased from Fisher Scientific. 3-Buten-1-amine was purchased from ABCR. For column chromatography, chloroform and hexane were purchased from CARLO ERBA reagents and dichloromethane, ethyl acetate, and ethanol were purchased from Reactolab. 21-Amino-4,7,10,13,16,19-hexaoxahenicosanoic acid was purchased from Iris Biotech. Dry *N*,*N′*-dimethylformamide, dry tetrahydrofuran, dry inhibitor free tetrahydrofuran, dry chloroform, dry acetonitrile, and silver fluoride were purchased from Acros Organics. Sodium bicarbonate, sodium chloride, sodium sulfate, and magnesium sulfate were purchased from Carl Roth. 4-(Dimethylamino)pyridinium-*p*-toluenesulfonate was prepared from 4-(dimethylamino)pyridin and *p*-toluenesulfonic acid as previously reported [36].

### 4.2. Nuclear Magnetic Resonance (NMR)

NMR spectroscopy was carried out on a Bruker Avance DPX 400 spectrometer at a temperature of 297.2 K with frequencies of 400.19 MHz for ^1^H nuclei and 100.63 MHz for ^13^C nuclei. The recorded spectra were calibrated to the residual solvent peak of DMSO-*d*_6_, CDCl_3_, or THF-*d*_8_. Data were analyzed with the MestReNova software suite (ver. 12.0) and chemical shifts *δ* are reported in parts per million (ppm) with coupling constant in Hz (multiplicity: s = singlet, d = doublet, dd = double doublet, t = triplet, m = multiplet, br = broad signal).

### 4.3. Mass Spectrometry (MS)

Mass spectrometry was carried out as service measurements by the Molecular and Biomolecular Analysis Service (MoBIAS) of ETH Zürich. High-resolution matrix-assisted laser desorption ionization (MALDI) mass spectrometry was carried out on a Bruker 9.4 T FT-ICR solariX using *trans*-2-[3-(4-*tert-*butylphenyl)-2-methyl-2-propenylidene]malononitrile (DCTB) as the matrix.

### 4.4. UV-Vis Absorption Spectroscopy

UV-vis absorption spectroscopy was carried out with a Shimazu UV-2401PC spectrophotometer. Quartz cuvettes (1 cm) were used.

### 4.5. Fluorescence Spectroscopy

Fluorescence spectroscopy measurements in solution and with solid samples were recorded with a Horiba Fluorolog 3 spectrometer with right angle illumination, which was equipped with a 450 W Xenon light source for excitation and a FL-1030-UP photomultiplier as the detector.

### 4.6. Differential Scanning Calorimetry (DSC)

DSC measurements were carried out on a Mettler Toledo DSC 2 STAR system under a nitrogen atmosphere with heating and cooling rates of 10 °C min^−1^ in a temperature range from −80 to 200 °C.

### 4.7. Thermogravimetric Analysis (TGA)

TGA measurements were conducted on a Mettler Toledo TGA/DSC 1 STAR system under ambient atmosphere in a temperature range from 25 to 600 °C and with a heating rate of 10 °C min^−1^.

### 4.8. Photographs

Photographs were recorded using a Nikon D7100 digital camera with an AF-S DX Zoom-NIKKOR 18–135 mm lens (f/3.5–5.6 G IF-ED).

### 4.9. Flash Column Chromatography

Column chromatography was performed with an Isolera One column chromatography system with a UV-vis detector.

### 4.10. Microwave Reactor

An Initiator8 microwave system equipped with an autosampler robot by Biotage was used for all microwave reactions.

### 4.11. Size-Exclusion Chromatography (SEC)

SEC measurements were carried out on an Agilent 1200-series HPLC system equipped with an Agilent PLgel mixed guard column (particle size = 5 µm) and two Agilent PLgel mixed-D columns (ID = 7.5 mm, L = 300 mm, particle size = 5 µm). Signal collection was performed with a miniDawn TREOS light scattering detector (Wyatt Technology Corp.), a UV detector (Agilent 1200 series), and an Optilab REX interferometric refractometer. THF was used as the eluent at a temperature of 30 °C with a flow rate of 1.0 mL min^−1^. The Astra software suite (Wyatt Technology Corp.) was used for data treatment; number-average molecular weights are provided in comparison with PS standards.

### 4.12. Protocol for the Preparation of **PU-D/A** Films

A sample of the polymer (0.2 g) was dissolved in THF (6.5 mL) over the course of 1 h and the solution was cast into a PTFE dish with a diameter of 6.5 cm. The film was placed under an inverse funnel for controlled evaporation and allowed to dry for 18 h under ambient conditions (23 °C) in a well-ventilated hood. The average film thickness of solvent-cast samples was determined as ca. 0.06 mm.

### 4.13. Emission Spectra of Polymer Films

Emission spectra of solid films of **PU-D/A** samples were recorded with an Ocean Optics USB 4000 spectrometer equipped with an Ocean Optics LS-450 LED light source and an Ocean Optics QR230-7-XSR SMA 905 optical fibre. Measurements were carried out with an excitation wavelength (*λ*_ex_) of 305 nm and an integration time of 5 s for each spectrum. Spectra of swollen samples of **PU-D/A** were recorded after immersion in the solvent for 4 h and placement of samples on a glass slide substrate immediately prior to recording the spectra.

### 4.14. Swelling Experiments

**PU-D/A** films (ca. 13–20 mg per sample) were immersed in either CHCl_3_ or MeOH for 5 h and solvent uptake was measured gravimetrically immediately after removal of the sample from the solvent-filled vial. For each solvent, three samples were prepared and the average solvent uptake was determined. The following equations were used to calculate the solvent uptake [37]:

$$m_{Solvent\;uptake}=m_{Swollen\;polymer}-m_{Dry\;polymer}$$

$$V_{Solvent\;uptake}=m_{Solvent\;uptake}/\rho_{Solvent}$$

$$Solvent\;uptake=V_{Solvent\;uptake}/m_{Dry\;polymer}$$

## Data Availability

The primary data generated and analyzed during this study are available from the Zenodo data repository at doi: 10.5281/zenodo.6577129 (accessed on 15 April 2022).

## Supplementary Materials

The following supporting information can be downloaded at https://www.mdpi.com/article/10.3390/gels8060350/s1, and includes Figures S1–S16, further experimental details [28,36], and the detailed chemical characterization data for all compounds.
