# Supplementary material for: Supramolecular Rings as Building Blocks for Stimuli-Responsive Materials
Source: Gels. 2022 Jun 3;8(6):350. doi: 10.3390/gels8060350 (PMC9222365; doi:10.3390/gels8060350)
Supplement: Supplementary file 1 [file gels-08-00350-s001.zip › gels-1730950-supplementary.pdf]

Supplementary Information

Supramolecular Rings as Building Blocks for Stimuli-Responsive Materials

Hanna Traeger,[a] Alyssa Ghielmetti,[a] Yoshimitsu Sagara,[b]

Stephen Schrettl,,[a] Christoph Weder*,[a]*

[a]Adolphe Merkle Institute, University of Fribourg

Chemin des Verdiers 4, 1700 Fribourg, Switzerland

[b]Department of Materials Science and Engineering, Tokyo Institute of Technology

2-12-1, Ookayama, Meguro-ku, Tokyo 152-8552, Japan

** To whom correspondence should be addressed:*

stephen.schrettl@unifr.ch

christoph.weder@unifr.ch

Table of Contents

1. Supplementary Figures S1–S16 3

2. Synthetic Procedures and Analytical Data 17

3. NMR Spectra Appendix 24

4. References 36

1. Supplementary Figures S1–S16

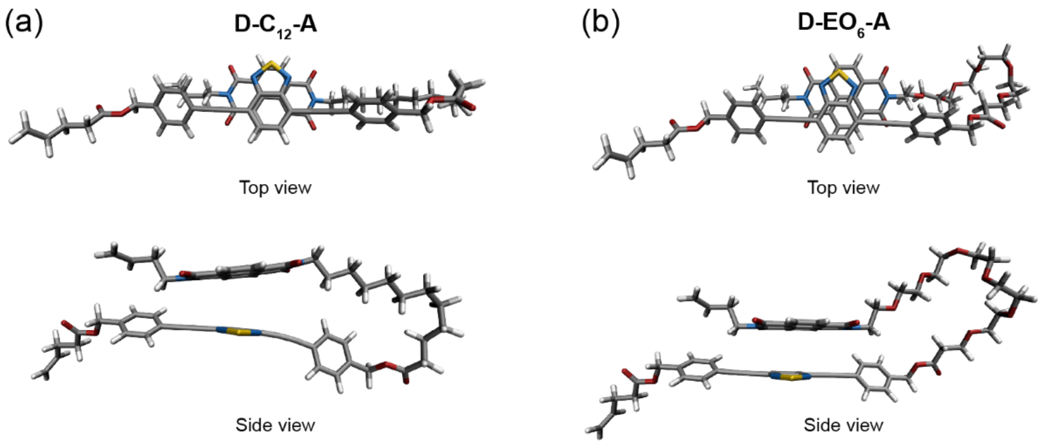

Supplementary Figure S1. Depiction of energy-minimized conformations of a loop-forming structure of a π-extended benzothiadiazole (emitter) and a naphthalene diimide (quencher) with (*a*) a C_{12} hydrocarbon linker and (*b*) an oligo(ethylene oxide) linker comprising six repeating units. In both cases, the structures were subjected to a universal force field (UFF) energy optimization. The displayed structures indicate that the dye interactions are not constrained by the length of the linker moiety.

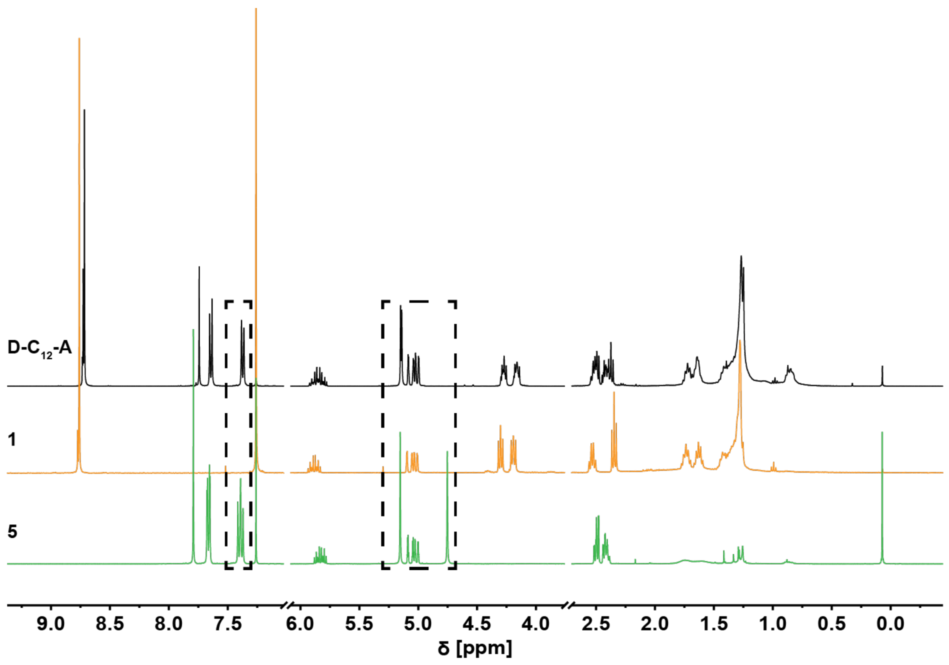

Supplementary Figure S2. Comparison of the ^{1}H-NMR spectra of **D-C$_{12}$-A**, and compounds **1** and **5**. The highlighted areas indicate changes in the signals associated with the aromatic protons of **5** (left box) as well as a down field shift of the signals corresponding to the methylene –CH_2-OH group after esterification (right box). The changes in the spectra indicate a successful linkage between **1** and **5**, resulting in the formation of the desired **D-C$_{12}$-A** motif.

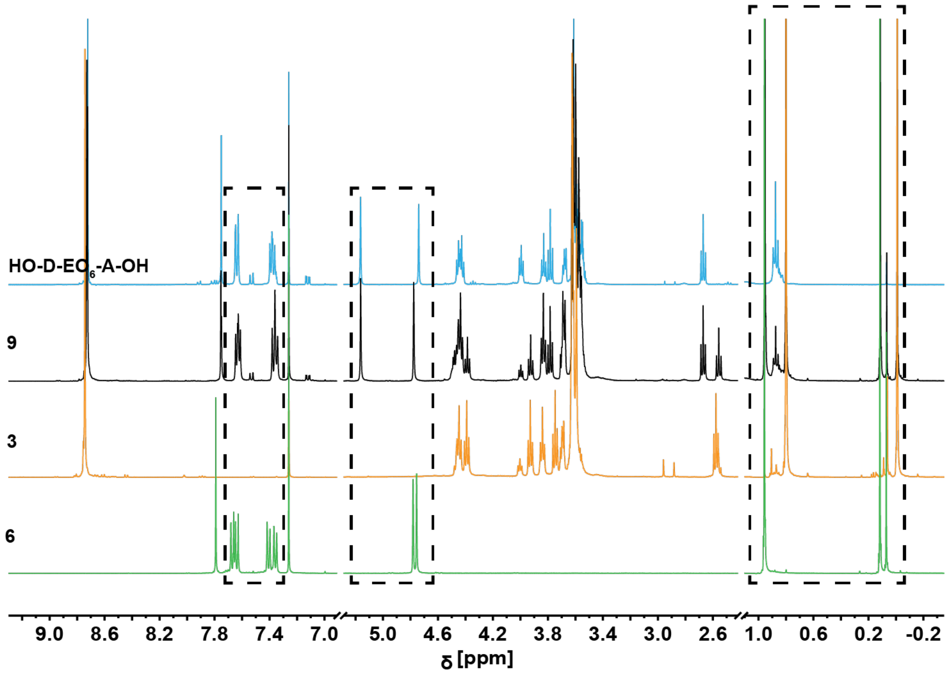

Supplementary Figure S3. Comparison of the ^{1}H-NMR spectra of **HO-D-EO$_6$-A-OH**, and the individual dyes **3** and **6**, and *tert*-butyltrimethylsilyl protected derivative **9**. The highlighted areas indicate changes in the signals associated with the aromatic protons of **5** (left box) as well as a down field shift of the signals corresponding to the methylene –C*H*$_2$-OH group after esterification (middle box) and the deprotection of **9** (right box). The changes in the spectra indicate a successful linkage between **3** and **6** and deprotection resulting in the formation of the desired **HO-D-EO$_6$-A-OH** motif.

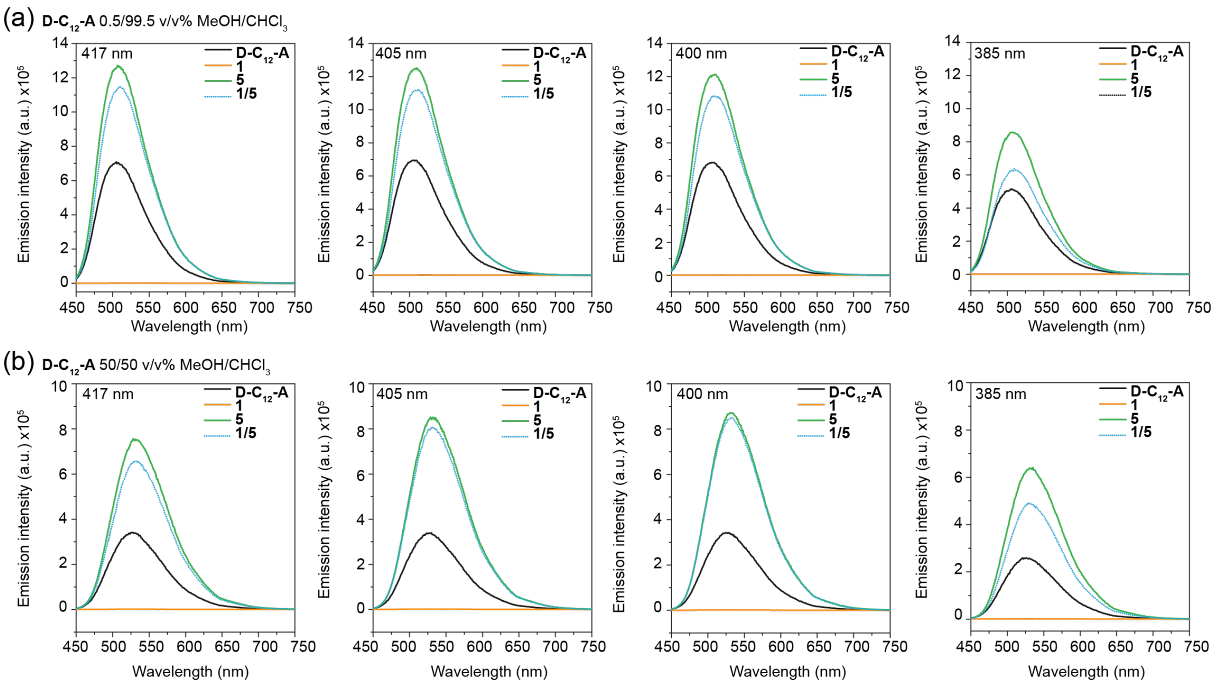

Supplementary Figure S4. Emission spectra of the **D-C$_{12}$-A** and the compounds **1** and **5** as well as equimolar mixture of **1/5** in mixtures of $CHCl_3$ and MeOH. (*a*) $CHCl_3$: MeOH = 99.5:0.5 v/v% (*b*) $CHCl_3$: MeOH = 50:50 v/v%. The concentration of all solutions was 10 μmol L^{-1}. An excitation wavelength (λ_{ex}) of either 417, 405, 400 or 385 nm was used, as indicated in the figure.

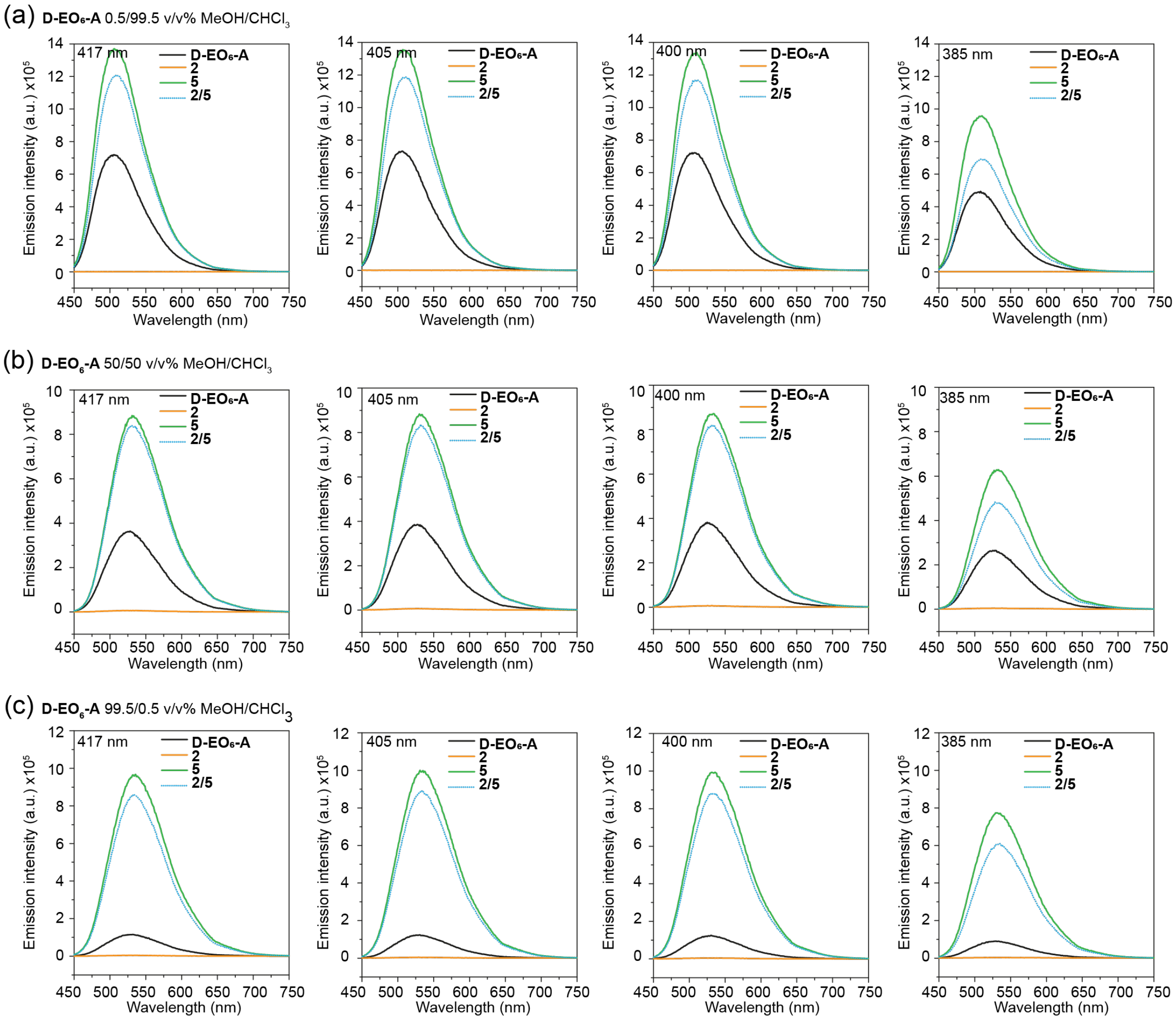

Supplementary Figure S5. Emission spectra of the **D-EO6-A** and the compounds **2** and **5** as well as equimolar mixture of **1/5** in mixtures of $CHCl_3$ and MeOH. (*a*) $CHCl_3$: MeOH = 99.5:0.5 v/v% (*b*) $CHCl_3$: MeOH = 50:50 v/v%. (*c*) $CHCl_3$: MeOH =0.5:99.5 v/v%. The concentration of all solutions was 10 μmol L^{-1}. An excitation wavelength (λ_{ex}) of either 417, 405, 400 or 385 nm was used, as indicated in the figure.

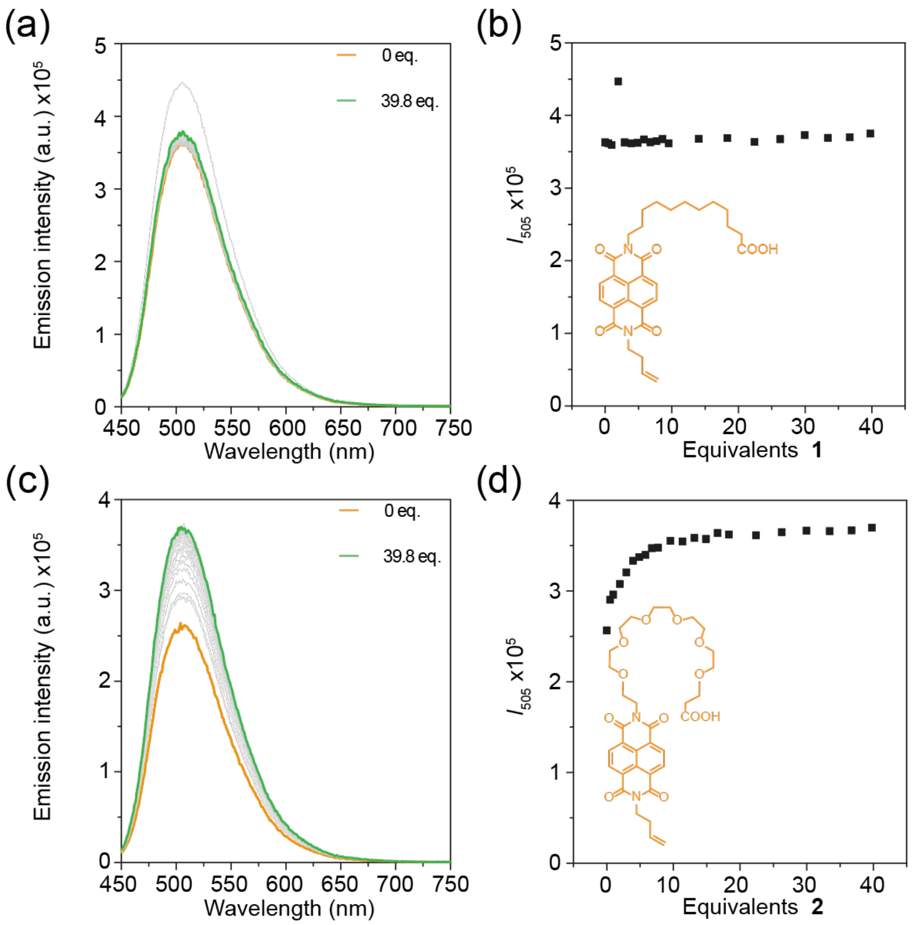

Supplementary Figure S6. Titrations between **5** and (*a*,*b*) **1** or (*c*,*d*) **2**. The titration was carried out by addition of aliquots of solutions of **1** or **2** (each 2500 µmol L^{-1}) to 2.5 mL of a solution of **5** (c = 2 µmol L^{-1}). To avoid dilution effects, the stock solutions of **1** and **2** (2500 µmol L^{-1}) in $CHCl_3$ already contained **5** (10 µmol L^{-1}). (*a*) Comparison of the emission spectra of **5** before (green) and after addition of 39.8 eq. of **1** (orange). (*b*) Plot of the maximum emission intensity of **5** at 505 nm as a function of the molar equivalents of **1** added. (*c*) Comparison of the emission spectra of **5** before (green) and after addition of 39.8 eq. of **2** (orange). (*d*) Plot of the maximum emission intensity of **5** at 505 nm as a function of the molar equivalents of **2** added.

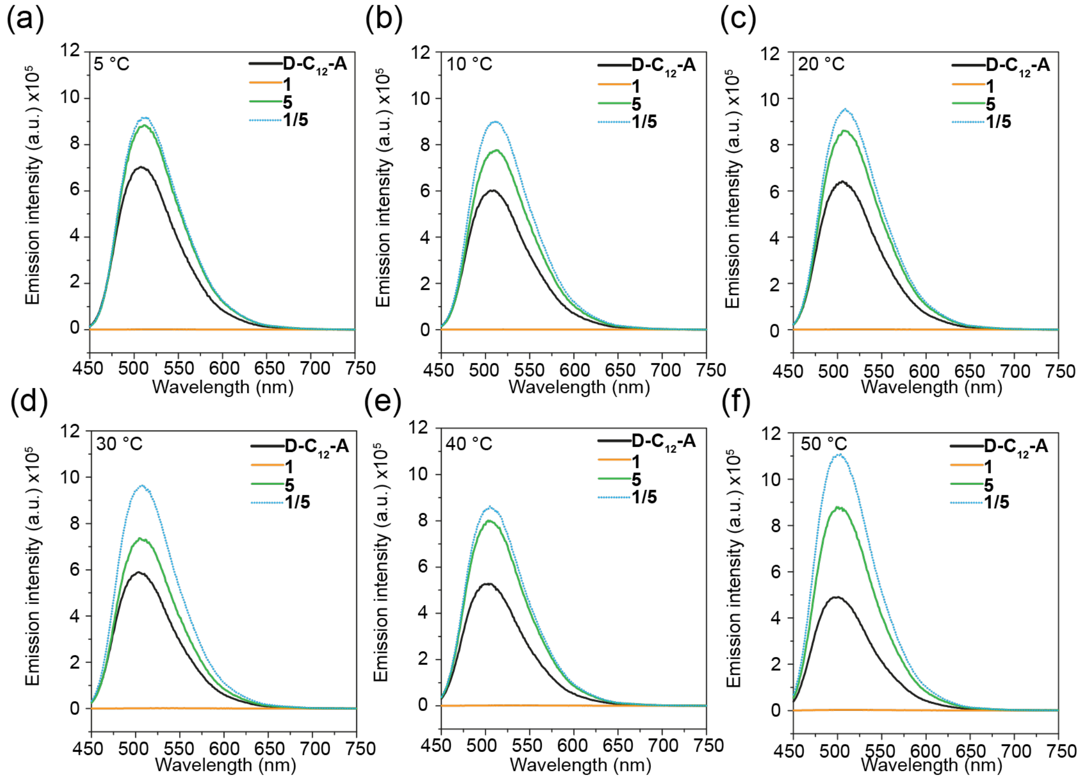

Supplementary Figure S7. Emission spectra of **D-C12-A 1, 5**, and an equimolar mixture of **1** and **5** in $CHCl_3$ (10 µmol L^{-1}). The spectra were recorded with an excitation wavelength (λ_{ex}) of 417 nm at (*a*) 5, (*b*) 10, (*c*) 20, (*d*) 30, (*e*) 40 and (*f*) 50 °C.

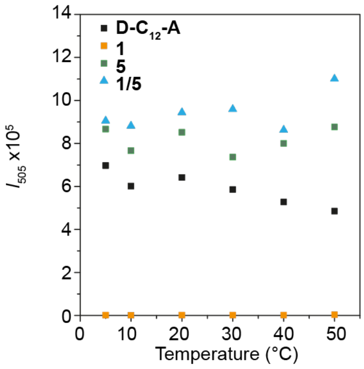

Supplementary Figure S8. Emission intensity at 505 nm of **D-C12-A** (black square), **1** (orange square), **5** (green square), and equimolar mixtures of **1** and **5** (blue triangle) in $CHCl_3$ solutions (10 µmol L^{-1}) as a function of temperature. Data were extracted from the spectra shown in **Figure S7**.

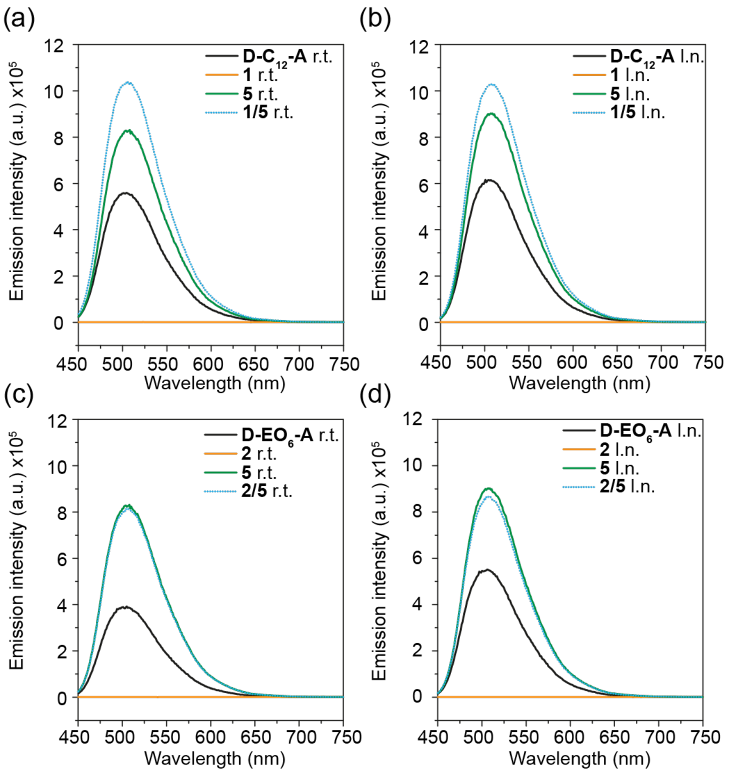

Supplementary Figure S9. Emission spectra of **D-C$_{12}$-A**, **1, 5**, and an equimolar mixture of **1** and **5** in $CHCl_3$ solutions recorded (*a*) at room temperature (r.t.) and (*b*) immediately after briefly immersing the cuvette in liquid nitrogen (l.n.). Emission spectra of the **D-EO$_6$-A**, **2, 5** and an equimolar mixture of **2** and **5** in $CHCl_3$ solutions recorded (*c*) at room temperature (r.t.) and (*d*) immediately after briefly immersing the cuvette in liquid nitrogen (l.n.). The concentration of all solutions was 10 μmol L^{-1} and an excitation wavelength (λ_{ex}) of 417 nm was used.

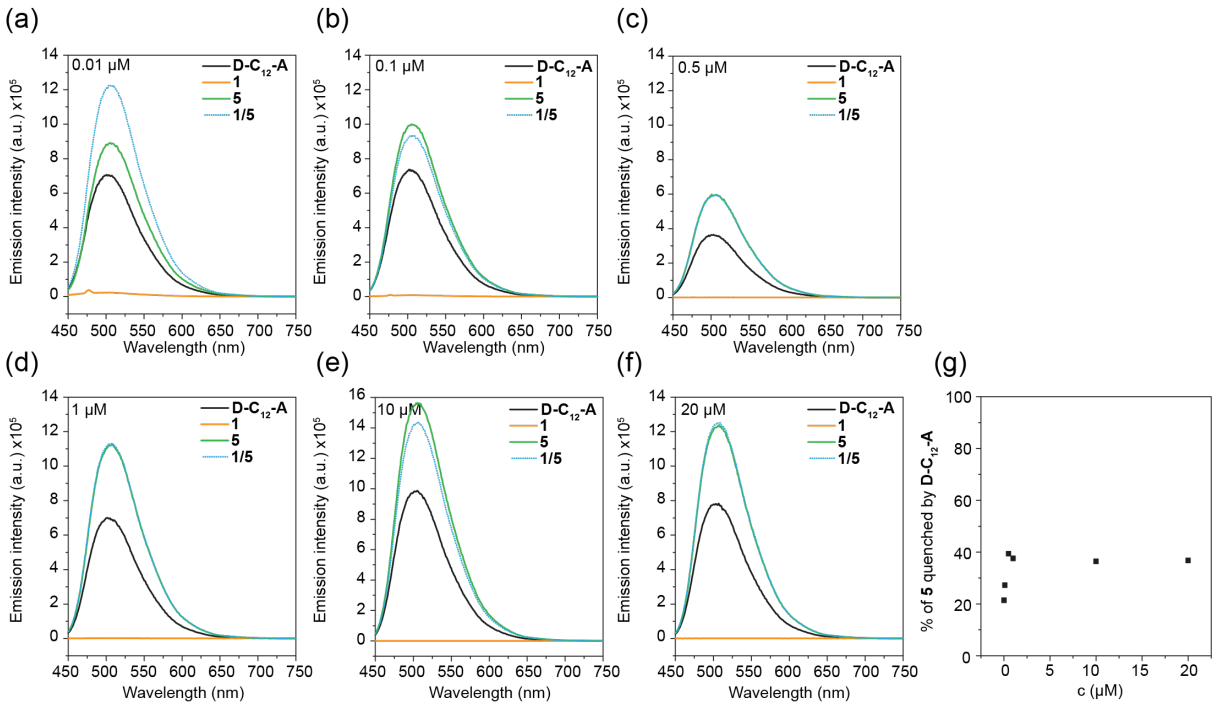

Supplementary Figure S10. Emission spectra of **D-C$_{12}$-A, 1, 5** and equimolar mixtures of **1** and **5** in $CHCl_3$ solutions with concentrations of (*a*) 0.01 µmol L^{-1}, (*b*) 0.1 µmol L^{-1}, (*c*) 0.5 µmol L^{-1}, (*d*) 1 µmol L^{-1}, (*e*) 10 µmol L^{-1}, and (*f*) 20 µmol L^{-1}. The spectra were recorded with an excitation wavelength (λ_{ex}) of 417 nm. (*g*) Plot of the percentage of quenching of the emission intensity at 505 nm observed for the **D-C$_{12}$-A** motif compared to the emission recorded for solutions of **5** as a function of the chromophore concentration.

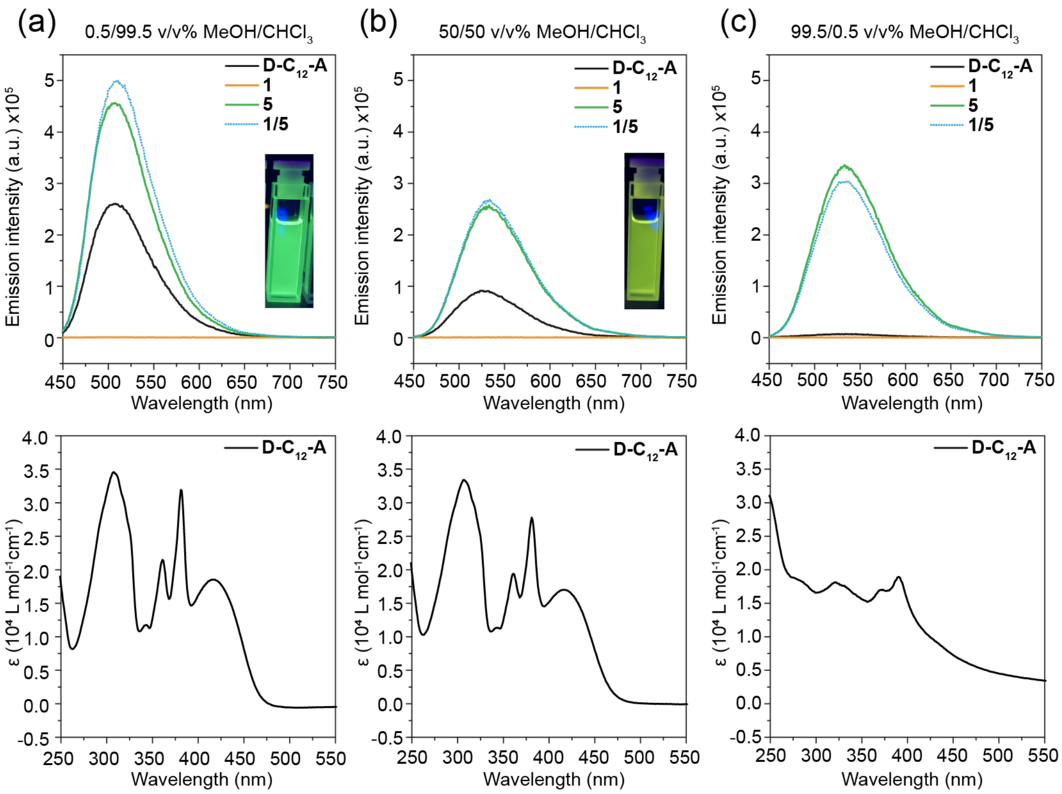

Supplementary Figure S11. Emission spectra (top) and UV-vis absorption spectra (bottom) of **D-C$_{12}$-A**, **1**, **5** and equimolar mixtures of **1/5** in MeOH/CHCl$_3$ solutions of (*a*) 0.5:99.5, (*b*) 50:50, and (*c*) 99.5:0.5 v/v%. The insets show photographs of the solutions that were illuminated by UV-light (365 nm). The concentration of all solutions was 10 μmol L^{-1} and an excitation wavelength (λ_{ex}) of 417 nm was used.

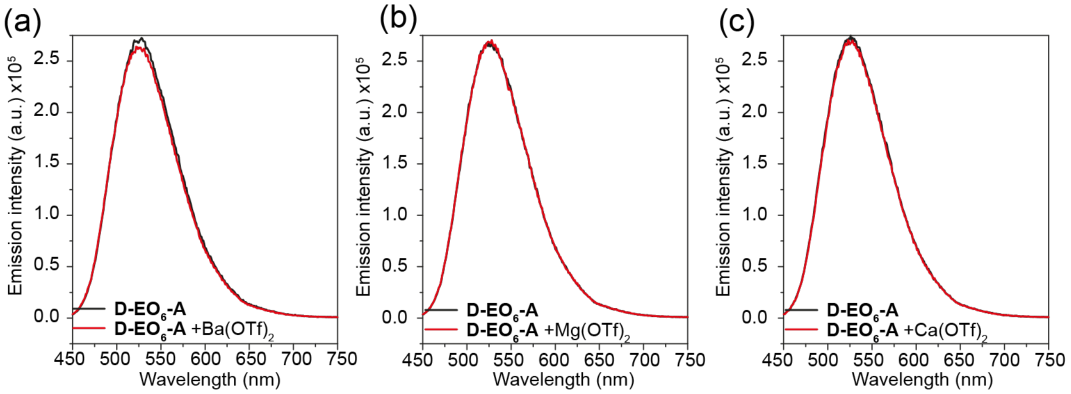

Supplementary Figure S12. Emission spectra (λ_{ex} = 417 nm) of solutions of **D-EO$_6$-A** (c = 10 µmol L^{-1} in MeOH/CHCl$_3$ 50:50 v/v%) before (black lines) and after (red lines) addition of 5 mg of (*a*) Ba(OTf)$_2$, (*b*) Mg(OTf)$_2$, and (*c*) Ca(OTf)$_2$.

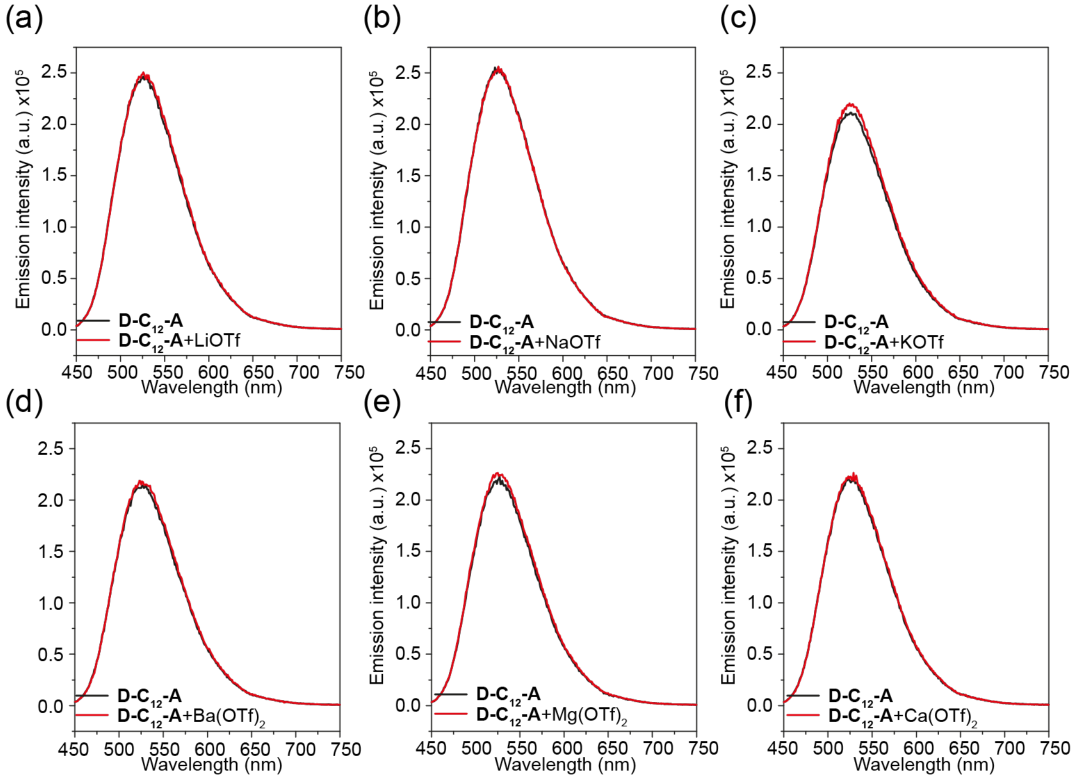

Supplementary Figure S13. Emission spectra (λ_{ex} = 417 nm) of solutions of **D-C$_{12}$-A** (c = 10 µmol L^{-1} in MeOH/CHCl$_3$ 50:50 v/v%) before (black lines) and after (red lines) addition of 5 mg of (*a*) LiOTf, (*b*) NaOTf, (*c*) KOTf, (*d*) Ba(OTf)$_2$, (*e*) Mg(OTf)$_2$, and (*f*) Ca(OTf)$_2$.

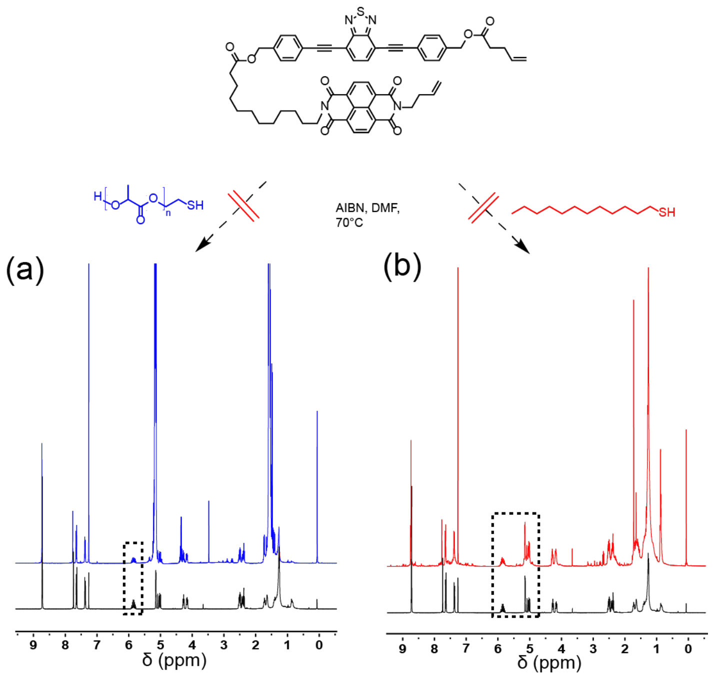

Supplementary Figure S14. Scheme showing the attempts toward a polymer incorporation of **D-C12-A** *via* thiol-ene click reactions with the depicted thiols. (*a*) The thiol-ene reaction of **D-C12-A** with thiol-terminated poly(lactic acid) (PLA-SH) was explored with AIBN as a radical source. Shown are the ^{1}H NMR spectrum of **D-C12-A** (black line) and the spectrum of the crude reaction mixture (blue line). (*b*) Thiol-ene reaction of **D-C12-A** with dodecyl thiol catalyzed by AIBN as a radical source. Shown are the ^{1}H NMR spectrum of **D-C12-A** (black line) and the spectrum of the crude reaction mixture (red line). All ^{1}H-NMR spectra were recorded in $CDCl_3$ (400 MHz, 297 K).

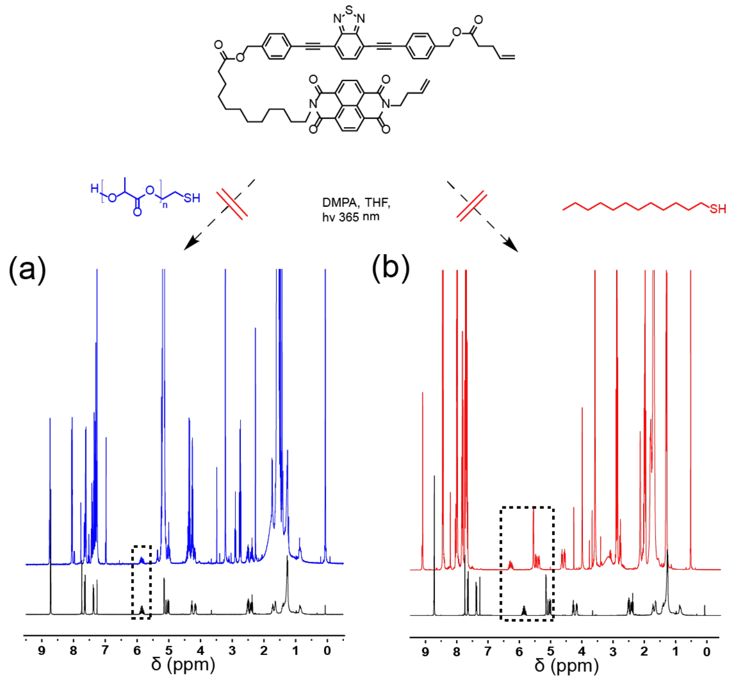

Supplementary Figure S15. Scheme showing the attempts toward a polymer incorporation of **D-C12-A** *via* thiol-ene click reaction with the depicted thiols. (*a*) Thiol-ene reaction of **D-C12-A** with a thiol-terminated poly(lactic acid) (PLA-SH) catalyzed by DMPA as a radical source. Shown is a comparison of the ^{1}H NMR spectrum of **D-C12-A** recorded before the reaction (black line) and the spectrum of the crude reaction mixture (blue line). (*b*) Thiol-ene reaction of **D-C12-A** with dodecyl thiol catalyzed by DMPA as a radical source. Shown is a comparison of the ^{1}H NMR spectrum of **D-C12-A** (black line) and the spectrum of the crude reaction mixture (red line). All ^{1}H-NMR spectra were recorded in $CDCl_3$ (400 MHz, 297 K).

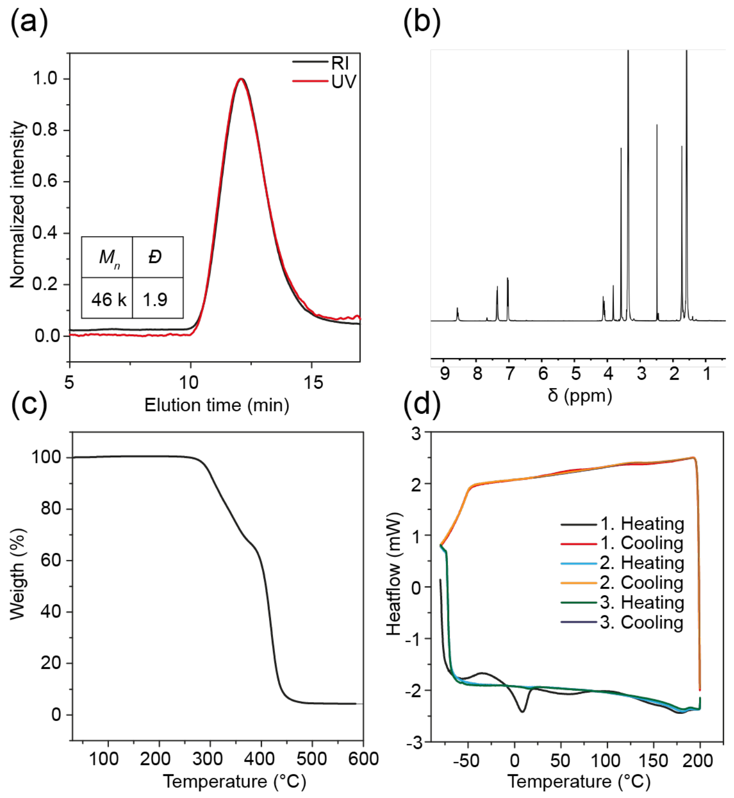

Supplementary Figure S16. (*a*) SEC traces of the polyurethane **PU-D/A** with the dRI (black line) and UV (red line) trace. (*b*) ^{1}H-NMR spectrum of **PU-D/A** recorded in THF-d_8 (400 MHz, 297 K). (*c*) Thermogravimetric analysis trace of a samples of **PU-D/A** recorded with a heating rate of 10 °C min^{-1}. (*d*) Comparison of the DSC heating and cooling traces for a **PU-D/A** sample in the temperature range from –80-200 °C recorded with a rate of 10 °C min^{-1}.

2. Synthetic Procedures and Analytical Data

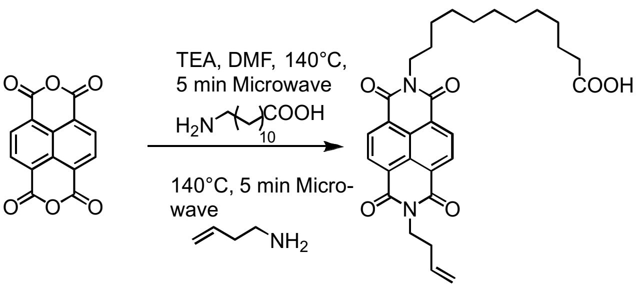

1

Synthesis of 1. The synthesis was adapted from a previous work by Tambara *et al.*[28] A mixture of naphthalene-1,4,5,8-tetracarboxylic dianhydride (500 mg, 1.74 mmol, 1 eq.), 12-aminolauric acid (376 mg, 1.74 mmol, 1 eq.) and (triethyl)amine (0.25 mL, 1.79 mmol, 1.05 eq.) was dissolved in DMF (20 mL). The solution was subjected to microwave irradiation for 5 min at 140 °C. 3-Buten-1-amine (168 µL, 1.79 mmol, 1.05 eq) was added and the solution was again subjected to microwave irradiation for 5 min at 140 °C. The solvent was removed *in vacuo* and the residue was suspended in acetone, precipitated in 1 M aq. HCl solution, and filtered. Purification of the filtrate by flash column chromatography (silica gel, chloroform/MeOH 98:2 v/v%) yielded **1** (400 mg; 47.8 %) as an off-white powder.

^{1}H NMR (400 MHz, $CDCl_3$): δ = 8.76 (s, 4H), 5.88 (ddt, *J* = 17.1, 10.1, 7.0 Hz, 1H), 5.18–4.94 (m, 2H), 4.30 (t, *J* = 7.3 Hz, 2H), 4.24–4.07 (m, 2H), 2.53 (q, *J* = 7.2 Hz, 2H), 2.35 (dd, *J* = 8.2, 6.7 Hz, 2H), 2.03–0.97 (m, 16H). ^{13}C NMR (101 MHz, $CDCl_3$): δ = 162.7, 134.5, 130.8, 130.8, 126.6, 126.4, 117.2, 40.8, 39.9, 33.3, 32.3, 29.2, 29.1, 29.0, 28.8, 27.9, 26.9, 24.5 (tertiary carbon atoms were not observed due to the limited solubility of **1**). MALDI-HRMS (m/z): calcd. for $C_{30}H_{35}N_2O_6$: 519.2490 (M+H$^+$); found: 519.2493.

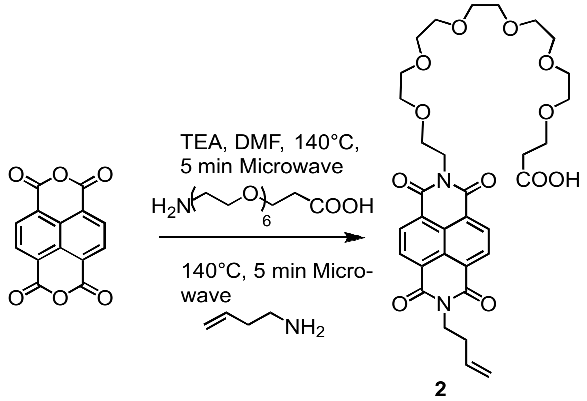

2

Synthesis of 2. The synthesis was adapted from a previous work by Tambara *et al.*[28] 21-Amino-4,7,10,13,16,19-hexaoxahenicosanoic acid (0.5 g, 1.4 mmol, 1 eq.), naphthalene-1,4,5,8-tetracarboxylic dianhydride (0.405 g, 1.4 mmol, 1 eq.), and (triethyl)amine (0.1 mL) were suspended in dry DMF (16 mL) in a 20 mL microwave vial. The mixture was sonicated for 2 min and subjected to microwave irradiation for 5 min at 140 °C. The reaction mixture was allowed to cool to room temperature, 3-buten-1-amine (0.129 mL, 0.1 g, 1.4 mmol, 1 eq.) was added, and the mixture was again subjected to microwave irradiation for

5 min at 140 °C. After removing the solvent *in vacuo*, the crude residue was resuspended in acetone and precipitated in 1M aqueous HCl. The filtrate was collected by centrifugation and dried. Flash column chromatography with a gradient elution from DCM to a mixture of 1:9 v/v% MeOH/DCM was performed to obtain **2** (334.7 mg, 37%) as an off-white solid.

^{1}H NMR (400 MHz, $CDCl_3$): δ = 8.65 (s, 4H), 5.81 (ddt, *J* = 17.1, 10.1, 7.0 Hz, 1H), 5.06–4.92 (m, 2H), 4.38 (t, *J* = 5.9 Hz, 2H), 4.21 (dd, *J* = 7.9, 6.8 Hz, 2H), 3.84–3.42 (m, 24H), 2.54 (t, *J* = 6.3 Hz, 2H), 2.50–2.42 (m, 2H). ^{13}C NMR (101 MHz, $CDCl_3$): δ = 174.2, 162.7, 162.6, 134.4, 130.8, 130.8, 130.7, 126.5, 126.5, 126.4, 126.3, 117.2, 70.4, 70.3, 70.3, 70.3, 70.2, 70.0, 69.9, 67.6, 66.3, 39.9, 39.4, 34.8, 32.2. MALDI-HRMS (m/z): calcd. for $C_{33}H_{40}N_2NaO_{12}$: 679.2473 (M+Na$^+$); found: 679.2476.

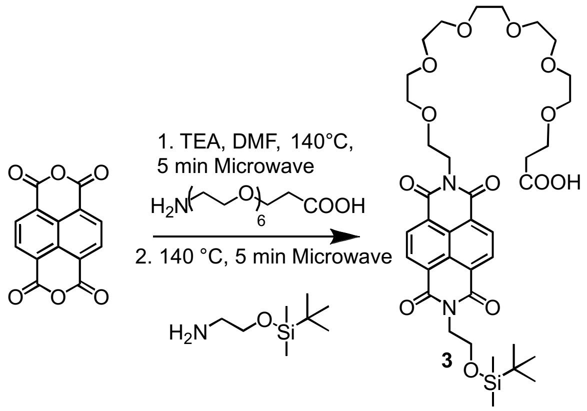

Synthesis of 3. The synthesis was adapted from a previous work by Tambara *et al.*[28] 21-Amino-4,7,10,13,16,19-hexaoxahenicosanoic acid (0.25 g, 0.7 mmol, 1 eq.), napthalene-1,4,5,8-tetracarboxylic dianhydrid (0.7 mmol, 1 eq), and (triethyl)amine (0.1 mL) were suspended in dry DMF (8 mL) in a 20 mL microwave vial. The mixture was sonicated for 2 min and subjected to microwave irradiation for 5 min at 140 °C. The reaction mixture was allowed to cool to room temperature, 2-((*tert*-butyldimethylsilyl)oxy)-ethanamine (0.118 mL, 0.124 g, 0.7 mmol, 1 eq.) was added, and the mixture was again subjected to microwave irradiation for 5 min at 140 °C. After removing the solvent *in vacuo*, the crude residue was resuspended in acetone, and precipitated in 1M aqueous HCl. The filtrate was collected by centrifugation and dried. Flash column chromatography with a gradient elution from DCM to a mixture of 2:8 v/v% MeOH/DCM was performed to obtain **3** (83.4 mg, 15.5%) as an off-white solid.

^{1}H NMR (400 MHz, $CDCl_3$): δ = 8.74 (s, 4H), 4.47–4.42 (m, 2H), 4.39 (t, *J* = 6.2 Hz, 2H), 3.93 (t, *J* = 6.2 Hz, 2H), 3.84 (td, *J* = 5.9, 2.4 Hz, 2H), 3.75 (t, *J* = 6.1 Hz, 2H), 3.70 (dd, *J* = 5.9, 3.5 Hz, 2H), 3.66–3.55 (m, 18H), 2.58 (t, *J* = 6.1 Hz, 2H), 0.80 (s, 9H), –0.01 (s, 6H).

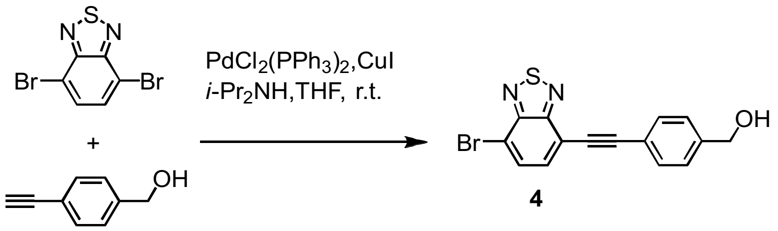

Synthesis of 4. A mixture of 4,7-dibromo-2,1,3-benzothiadiazole (1.5 g, 5.11 mmol, 1 eq.), $PdCl_2(PPh_3)_2$ (182 mg, 0.25 mmol, 0.05 eq.), CuI (53 mg, 0.25 mmol, 0.05 eq.), and (diisopropyl)amine (0.716 mL, 5.11 mmol, 1 eq.) was dissolve in THF (60 mL). The mixture was cooled to 0 °C and 4-ethynylbenzyl alcohol (675 mg, 5.11 mmol, 1 eq.) in THF (40 mL) was added dropwise over 15 min. The reaction mixture was allowed to warm to room temperature and stirred for 1 h. The mixture was then poured into ethyl acetate (200 mL), washed with 5% aq. HCl solution (3 × 100 mL), sat. aq. $NaHCO_3$ solution (100 mL), and sat. aq. NaCl solution (150 mL). The organic layer was dried over $MgSO_4$, filtered, the solvent was removed *in vacuo*, and purification by flash column chromatography (silica gel, hexanes/ethyl acetate 4:3 v/v%) yielded **4** (539 mg, 1.55 mmol, 31%) as a bright orange solid.

^1H NMR (400 MHz, DMSO-d_6): δ = 8.05 (d, J = 7.6 Hz, 1H), 7.83 (d, J = 7.6 Hz, 1H), 7.63–7.56 (m, 2H), 7.42 (d, J = 8.0 Hz, 2H), 5.32 (t, J = 5.7 Hz, 1H), 4.56 (d, J = 5.7 Hz, 2H). ^{13}C NMR (100 MHz, DMSO-d_6): δ = 153.5, 152.4, 144.3, 133.2, 132.3, 131.3, 126.7, 119.8, 115.5, 114.1, 96.2, 84.6, 62.5. MALDI-HRMS (m/z): calcd. for $C_{15}H_{10}BrN_2OS$: 344.9692 (M+H$^+$); found: 344.9692.

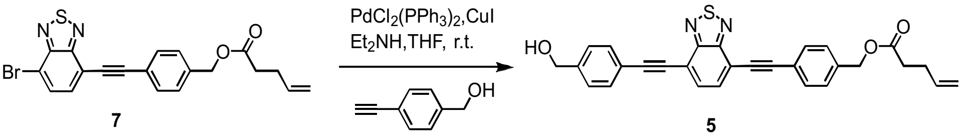

Synthesis of 5. 4-(4-(Ethynylbenzyl)-1-(pent-4-enoate))-7-bromo-2,1,3-benzothiadiazole **7** (129 mg, 0.3 mmol, 1 eq.), $PdCl_2(PPh3)_2$ (11 mg, 0.015 mmol, 0.05 eq.), CuI (3 mg, 0.015 mmol, 0.05 eq.) and dry (diethyl)amine (37 µL, 0.36 mmol, 1.2 eq) were dissolve in dry THF (2 mL) and a solution of 4-ethynylbenzyl alcohol (50 mg, 0.36 mmol, 1.2 eq.) in dry THF (1 mL) was added dropwise. The mixture was stirred for 2.5 h and then poured into ethyl acetate (50 mL), washed with 5% aq. HCl solution (3 × 25 mL), sat. aq. $NaHCO_3$ solution (30 mL), and sat. aq. NaCl solution (30 mL). The organic layer was dried over $MgSO_4$, filtered, and the solvent was removed *in vacuo*. The crude product was purified by flash column chromatography (silica gel, DCM/MeOH 98:2 v/v%) to yield **5** (139 mg, 97%) as a red solid.

^1H NMR (400 MHz, DMSO-d_6): δ = 7.98–7.90 (m, 2H), 7.67–7.59 (m, 4H), 7.48–7.41 (m, 4H), 6.04–5.61 (m, 1H), 5.33 (t, J = 5.7 Hz, 1H), 5.15 (s, 2H), 5.11–4.88 (m, 2H), 4.57 (d, J = 5.6 Hz, 2H), 2.37–2.27 (m, 2H). ^{13}C NMR (100 MHz, DMSO-d_6): δ = 172.1, 153.7, 153.7, 144.4, 137.7, 136.9, 132.9, 132.7, 131.6, 131.4, 128.2, 126.7, 121.2, 119.8, 116.4, 115.9, 115.6, 97.2, 96.5, 85.9, 85.2, 64.9, 62.5, 32.7, 28.4. MALDI-HRMS (m/z): calcd. for $C_{29}H_{22}N_2SO_3$: 478.1346 (M$^+$); found: 478.1350.

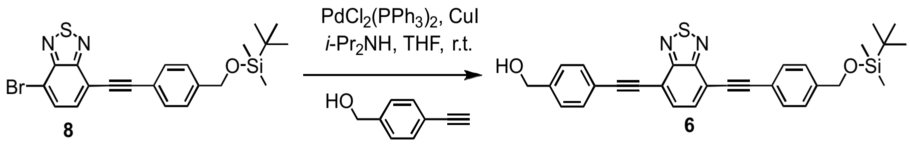

8 **6**

Synthesis of 6. 4-Bromo-7-((4-(((*tert*-butyldimethylsilyl)oxy)methyl)phenyl)ethynyl)benzo[c][1,2,5]thiadiazole **8** (250 mg, 0.54 mmol, 1 eq.), $PdCl_2(PPh_3)_2$ (190 mg, 0.272 mmol, 0.05 eq.), CuI (51 mg, 0.272 mmol, 0.05 eq.), and (diisopropyl)amine (95 µL, 0.65 mmol, 1.2 eq) were dissolve in dry THF (4 mL). 4-Ethynylbenzyl alcohol (86 mg, 0.65 mmol, 1.2 eq.) in dry THF (2 mL) was added dropwise. The mixture was stirred for 2.5 h and poured into ethyl acetate (50 mL), washed with 5% aq. HCl solution (3 × 25 mL), sat. aq. $NaHCO_3$ solution (30 mL), and sat. aq. NaCl solution (30 mL). The organic layer was dried over $MgSO_4$, filtered, and the solvent was removed *in vacuo*. The crude product was purified by flash column chromatography (silica gel, DCM to DCM /EtOAc 9:1 v/v%) to yield **6** (139 mg, 50%) as a yellow solid.

^1H NMR (400 MHz, $CDCl_3$): δ = 7.79 (s, 1H), 7.70–7.60 (m, 2H), 7.38 (ddq, *J* = 19.8, 7.5, 0.8 Hz, 2H), 4.78 (s, 1H), 4.76 (s, 1H), 0.96 (s, 5H), 0.12 (s, 3H). ^{13}C NMR (101 MHz, $CDCl_3$): δ = 153.4, 153.4, 141.8, 140.9, 134.1, 134.0, 134.0, 131.5, 131.3, 131.2, 130.9, 129.5, 127.1, 127.0, 127.0, 125.8, 125.0, 120.7, 119.9, 116.4, 116.0, 96.8, 96.3, 84.4, 84.0, 28.7, 24.9, 17.4, 0.0, –6.3.

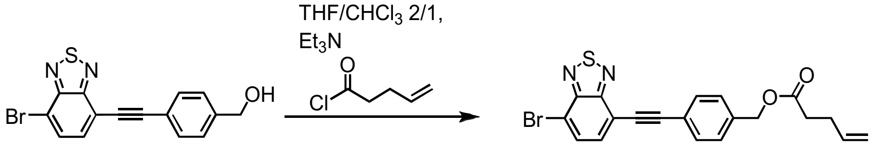

4 **7**

Synthesis of 7. 4-(4-Ethynylbenzyl alcohol)-7-bromo-2,1,3-benzothiadiazole **4** (200 mg, 0.57 mmol, 1 eq.) and dry (triethyl)amine (89 µL, 0.86 mmol, 1.5 eq.) were dissolve in dry THF (6.5 mL) and dry chloroform (3 mL). 4-Pentenoyl chloride (0.127 mL, 1.15 mmol, 2 eq.) was added and the reaction mixture was stirred for 3 d. The reaction mixture was poured into ethyl acetate (100 mL), washed with 1 M aq. HCl solution (4 × 50 mL), sat. aq. $NaHCO_3$ solution (60 mL), and sat. aq. NaCl solution (60 mL). The organic layer was dried over $MgSO_4$, filtered, the solvent was removed *in vacuo*. The crude product was purified by flash column chromatography (silica gel, hexanes/DCM 2:1 v/v%) to yield **7** (178 mg, 0.42 mmol, 72%) as a yellow solid.

^1H NMR (400 MHz, $CDCl_3$): δ = 7.83 (d, *J* = 7.6 Hz, 1H), 7.70–7.60 (m, 3H), 7.38 (d, *J* = 8.5 Hz, 2H), 5.82 (ddt, *J* = 16.4, 10.2, 6.3 Hz, 1H), 5.14 (s, 2H), 5.06 (dq, *J* = 17.1, 1.6 Hz, 1H), 5.00 (dq, *J* = 10.2, 1.4 Hz, 1H), 2.49 (ddd, *J* = 7.8, 6.7, 1.6 Hz, 2H), 2.47–2.35 (m, 2H). ^{13}C NMR (100 MHz, $CDCl_3$): δ = 172.8, 154.3, 153.2, 137.2, 136.6, 133.0, 132.2, 132.1, 128.2, 122.4, 116.7, 115.8, 114.9, 96.6, 85.1, 65.8, 33.6, 28.9.

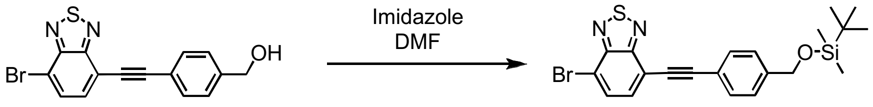

4 **8**

Synthesis of 8. 4-(4-Ethynylbenzyl alcohol)-7-bromo-2,1,3-benzothiadiazole **4** (0.1 g 0.282 mmol, 1eq.) as dissolved in dry DMF and imidazole (0.0288 g, 0.42 mmol, 1.5 eq.) was added. The reaction mixture was cooled in an ice bath and *tert*-butyldimethylsilyl chloride (0.051, 0.34 mmol, 1.2 eq.) dissolved in DMF was

added dropwise. The reaction mixture was allowed to warm to room temperature and stirred overnight, poured into distilled water, and the mixture was extracted with $CHCl_3$. The organic phases were combined, washed with brine, with water, and dried over Na_2SO_4. The crude product was purified by flash column chromatography (silica gel, hexanes/DCM 1:1 v/v%) to yield **8** (50 mg, 39%) as a yellow solid.

^{1}H NMR (400 MHz, $CDCl_3$): δ = 7.84 (d, J = 7.6 Hz, 2H), 7.69–7.58 (m, 3H), 7.35 (dq, J = 7.4, 0.8 Hz, 2H), 4.77 (s, 2H), 0.95 (s, 10H), 0.11 (s, 6H). ^{13}C NMR (101 MHz, $CDCl_3$): δ = 154.3, 153.2, 142.9, 132.8, 132.1, 126.1, 120.9, 117.0, 114.6, 97.3, 84.4, 64.8, 26.1, 18.6, –5.1.

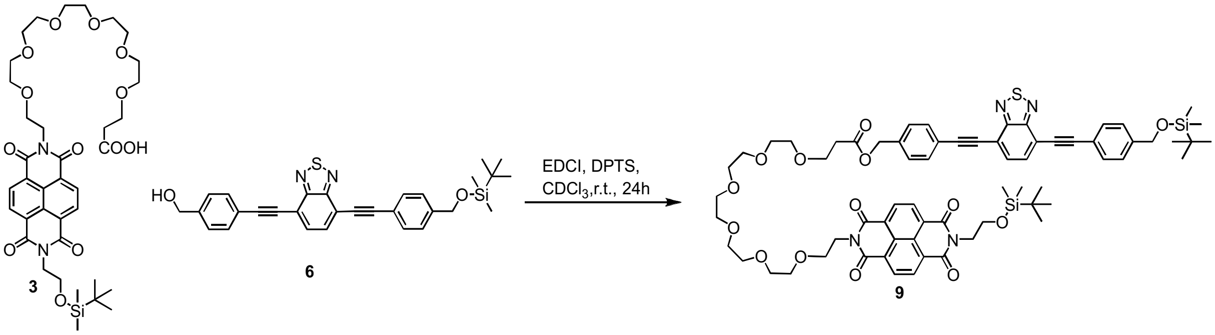

Synthesis of 9. Compound **3** (0.083 g, 0.109 mmol, 1.2 eq), 1-ethyl-3-(3-dimethylaminopropyl)carbodiimid hydrochloride (EDCI, 0.034 g, 0.127 mmol, 1.4 eq.), and 4-(dimethylamino)pyridinium-*p*-toluenesulfonate[36] (DPTS, 0.010 g, 0.0363 mmol, 0.4 eq) were dissolved in dry chloroform (5.5 mL) under nitrogen atmosphere. Compound **6** (0.037 g, 0.0908 mmol, 1 eq.) was added and the mixture was stirred at room temperature for 18 h. The mixture was transferred to a separatory funnel and the organic phase was washed three times with saturated NaCl solution, dried over Na_2SO_4, and filtered. The crude product was purified by flash column chromatography (silica gel, gradient eluent: DCM to DCM/MeOH 9:1 v/v%) to obtain **9** (62.5 mg, 56%) as a yellow-brown solid.

^{1}H NMR (400 MHz, $CDCl_3$): δ = 8.72 (s, 4H), 7.75 (s, 2H), 7.67–7.52 (m, 4H), 7.44–7.30 (m, 4H), 5.16 (s, 2H), 4.77 (s, 2H), 3.92 (t, J = 6.2 Hz, 2H), 3.83 (t, J = 6.0 Hz, 2H), 3.78 (t, J = 6.4 Hz, 2H), 3.71–3.66 (m, 2H), 3.63–3.56 (m, 18H), 2.67 (t, J = 6.4 Hz, 2H), 0.95 (s, 9H), 0.80 (s, 10H), 0.11 (s, 6H), –0.01 (s, 6H). ^{13}C NMR (101 MHz, $CDCl_3$): δ = 162.8, 162.7, 162.6, 162.6, 154.1, 146.9, 142.6, 136.8, 132.3, 132.1, 131.9, 131.7, 130.8, 130.7, 128.4, 128.3, 127.8, 126.6, 126.4, 126.4, 125.8, 124.3, 123.8, 122.2, 120.7, 117.3, 116.7, 97.7, 96.8, 85.5, 84.8, 70.4, 70.4, 70.3, 70.3, 69.9, 67.6, 66.4, 65.6, 64.5, 61.3, 60.9, 59.5, 42.1, 39.4, 34.9, 31.3, 30.0, 29.5, 25.8, 25.6, 22.5, 18.2, 18.0, 13.9, 0.8, –3.8, –5.42, –5.6.

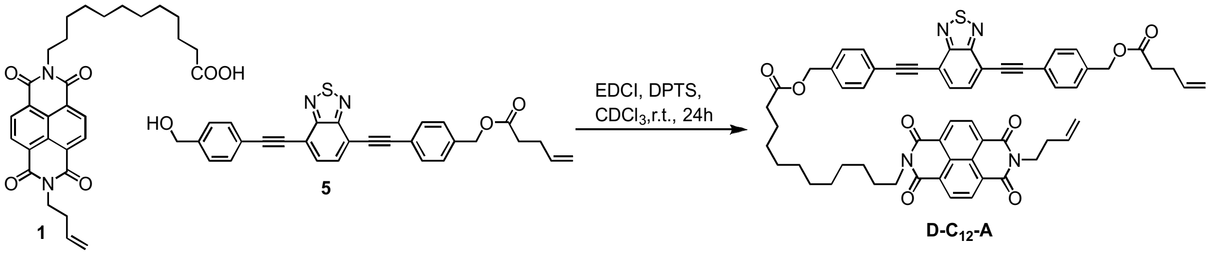

Synthesis of D-C$_{12}$-A. *N*-(4-Butylen)-*N'*-(12-dodecanoic acid)naphthalene-1,4,5,8-tetracarboxylic diimide **1** (108 mg, 0.2 mmol, 2 eq.), EDCI (49 mg, 0.25 mmol, 2.4 eq.), and DPTS (14 mg, 0.04 mmol, 0.4 eq.) were dissolved in dry chloroform (3 mL). **5** (50 mg, 0.10 mmol, 1 eq) was added and the mixture was stirred for

16 h at room temperature. The mixture was then poured into chloroform (10 mL) and washed with sat. aq. NaCl solution (3 × 10 mL). The organic layer was dried over $MgSO_4$, filtered, and the solvent was removed *in vacuo*. Purification by flash column chromatography (silica gel, gradient eluent: DCM to DCM/MeOH 98.5:1.5 v/v%) yielded **D-C$_{12}$-A** (70 mg, 0.07 mmol, 72%) as a yellow solid.

^{1}H NMR (400 MHz, $CDCl_3$): δ = 8.72 (s, 4H), 7.75 (s, 2H), 7.66 (d, J = 8.1 Hz, 4H), 7.38 (d, J = 7.9 Hz, 4H), 5.85 (tdt, J = 16.5, 10.2, 6.6 Hz, 2H), 5.15 (s, 2H), 5.14 (s, 2H), 5.11–4.97 (m, 4H), 4.27 (t, J = 7.3 Hz, 2H), 4.21–4.12 (m, 2H), 2.57–2.45 (m, 4H), 2.47–2.33 (m, 4H), 1.77–1.62 (m, 6H), 1.48–1.30 (m, 14H), 1.40 (s, 4H (HDO). ^{13}C NMR (100 MHz, $CDCl_3$): δ = δ 173.7, 172.9, 169.79, 162.9, 162.9, 154.4, 137.4, 137.2, 136.6, 134.8, 132.6, 132.3, 131.1, 131.1, 131.0, 131.0, 128.2, 128.2, 126.8, 126.9, 126.8, 126.6, 126.6, 122.4, 122.4, 117.5, 117.3, 117.2, 115.8, 97.3, 97.3, 85.8, 85.7, 65.8, 65.6, 41.1, 40.2, 35.4, 34.4, 33.7, 32.6, 32.1, 29.8, 29.6, 29.5, 29.5, 29.4, 29.4, 29.3, 29.3, 29.2, 29.0, 29.0, 28.2, 27.2, 25.1, 24.4, 22.8, 20.5, 14.3. MALDI-HRMS (m/z): calcd. for $C_{59}H_{54}N_4SO_8$: 978.3657 (M^+); found: 978.3661.

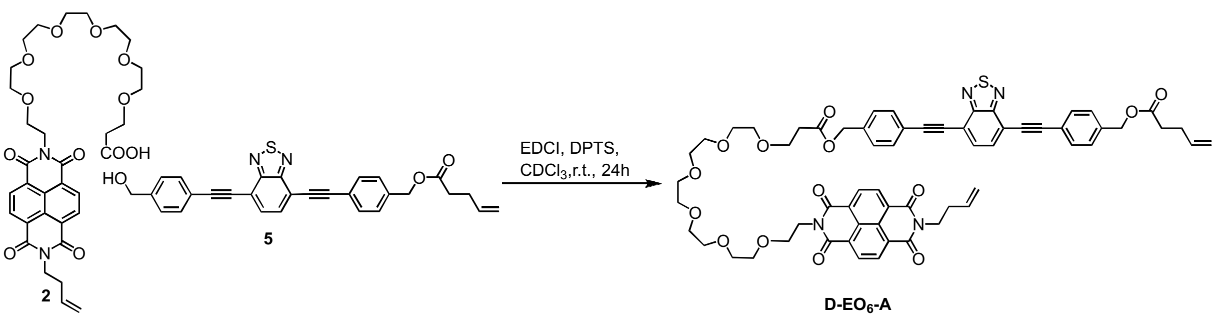

Synthesis of D-EO$_6$-A. Compound **2** (96.4 mg, 0.146 mmol, 1.0 eq.), EDCI (0.028 g, 0.146 mmol, 1.0 eq.), and DPTS (0.031 g, 0.146 mmol, 1,0eq.) were dissolved in dry chloroform (4 mL). **5** (0.049 g, 0.1 mmol, 0.7 eq.) was separately dissolved in chloroform (4 mL) and the solution was added to the mixture containing **2.** After stirring for 12 h at room temperature, thin layer chromatography indicated an incomplete reaction and an additional solution containing EDCI (10 mg, 0.05 mmol) and DPTS (5 mg, 0.02 mmol) was added. After 5 h, the mixture was transferred to a separatory funnel and washing three times with saturated NaCl solution, and dried over sodium sulfate. The crude residue was purified twice by column chromatography (silica gel, gradient eluent: DCM to DCM/MeOH 9:1 v/v%) to obtain **D-EO$_6$-A** (0.030 mg, 17%) as a yellow solid.

^{1}H NMR (400 MHz, $CDCl_3$): δ = 8.73 (s, 4H), 7.77 (s, 2H), 7.72–7.56 (m, 4H), 7.46–7.31 (m, 4H), 6.00–5.72 (m, 2H), 5.16 (d, J = 4.6 Hz, 4H), 5.11–4.93 (m, 4H), 4.44 (t, J = 5.9 Hz, 2H), 4.35–4.20 (m, 2H), 3.81 (dt, J = 18.7, 6.1 Hz, 4H), 3.72–3.53 (m, 19H), 2.67 (t, J = 6.4 Hz, 2H), 2.59–2.46 (m, 4H), 2.45–2.36 (m, 2H). ^{13}C NMR (101 MHz, $CDCl_3$): δ = 173.2, 171.8, 163.3, 163.2, 137.5, 137.5, 137.0, 135.1, 132.9, 132.6, 132.6, 131.4, 128.6, 128.5, 127.2, 127.1, 127.1, 127.0, 122.8, 122.8, 117.8, 117.6, 116.1, 97.6, 86.1, 71.1, 71.1– 71.0 (m), 71.0, 71.0, 70.9, 70.6, 68.3, 67.0, 66.2, 66.1, 40.5, 40.0, 35.6, 34.0, 32.9, 29.3. MALDI-HRMS (m/z): calcd. for $C_{62}H_{60}N_4NaO_{14}S$: 1139.3719 ($M+Na^+$); found: 1139.3714.

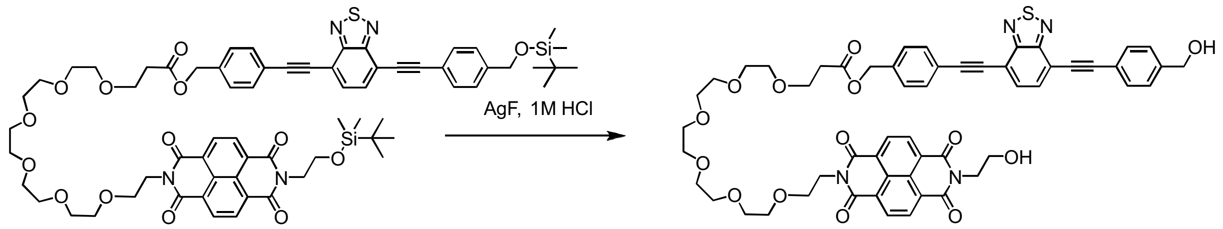

9 **HO-D-EO$_6$-A-OH**

Synthesis of HO-D-EO$_6$-A-OH. Compound **9** (40 mg, 0.0327 mmol, 1 eq.) was dissolved in dry acetonitrile (3 mL) and silver fluoride (7 mg, 0.055 mmol, 1.68 eq.) was added. The reaction mixture was cooled in an ice bath and 1M HCl (aq., 5mL) was added. The mixture was stirred for 3 h and poured into $CHCl_3$. The organic phase was washed three times with 1M HCl, two times with saturated aqueous NaCl solution, and dried over sodium sulfate. The crude product was purified by column chromatography (silica gel, gradient eluent: DCM to DCM/MeOH 9:1 v/v%) to yield **HO-D-EO$_6$-A-OH** (33 mg, 98%) as a brown-yellow solid.

^{1}H NMR (400 MHz, $CDCl_3$): δ = 8.72 (s, 4H), 7.75 (s, 2H), 7.69–7.58 (m, 4H), 7.38 (ddd, J = 9.3, 5.7, 2.7 Hz, 4H), 5.16 (s, 2H), 4.74 (s, 2H), 4.49–4.39 (m, 4H), 3.99 (dd, J = 5.9, 4.9 Hz, 2H), 3.83 (t, J = 5.9 Hz, 2H), 3.78 (t, J = 6.4 Hz, 2H), 3.68 (dd, J = 6.0, 3.6 Hz, 2H), 3.63–3.52 (m, 18H), 2.67 (t, J = 6.4 Hz, 2H). ^{13}C NMR (101 MHz, $CDCl_3$): δ = 171.5, 163.7, 162.9, 154.4, 142.2, 137.1, 132.6, 132.5, 132.3, 132.3, 131.3, 131.1, 128.2, 127.0, 126.9, 126.8, 126.5, 124.6, 124.1, 122.5, 121.7, 117.4, 117.1, 97.6, 97.3, 85.8, 85.4, 70.8, 70.7, 70.7, 70.7, 70.6, 70.6, 70.3, 67.9, 66.7, 65.9, 65.0, 61.3, 43.1, 39.8, 35.3, 35.0, 34.7, 32.1, 31.6, 30.3, 29.8, 29.8, 29.5, 22.8, 14.3.

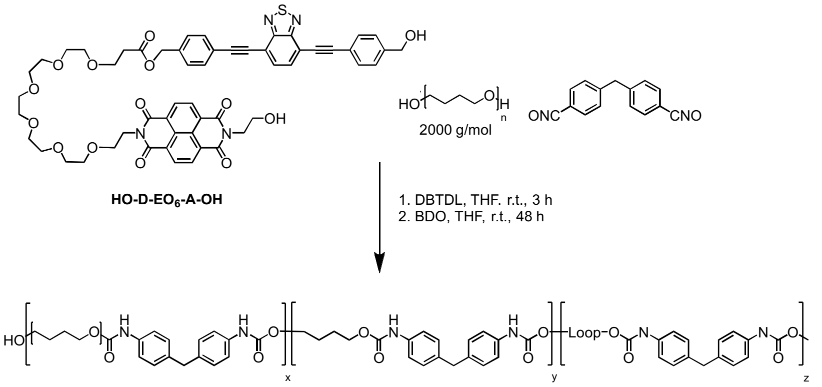

PU-D/A

Synthesis of PU-D/A. Dried poly(tetra hydrofuran) (3.0 g, 1.5 mmol, M_n = 2000 g mol^{-1}), **HO-D-EO$_6$-A-OH** (7.0 mg, 6.9 µmol), and 4,4′-methylenebis(phenylisocyanate) (1.31 g, 5.2 mmol) were added into a Schlenk flask. The mixture was dissolved in dry, inhibitor free THF (15 mL) and two drops of dibutyltin dilaurate (DBTDL) were added. After stirring for 3 h at room temperature, 1,4-butanediol (0.315 g, 3.5 mmol) was added dropwise to the solution. The mixture was stirred for 48 h and quenched by addition of MeOH. After precipitation in ethanol and hexane, **PU-D/A** (3.85 g, 83%) was obtained as a yellow polymer.

^{1}H NMR (400 MHz, THF-d_8): δ = 8.56 (d, J = 11.2 Hz, 1H), 7.36 (dd, J = 8.3, 2.5 Hz, 2H), 7.03 (d, J = 8.1 Hz, 3H), 4.10 (dd, J = 13.1, 6.4 Hz, 2H), 3.36 (d, J = 5.4 Hz, 19H), 1.58 (p, J = 2.9 Hz, 18H).

3. NMR Spectra Appendix

^{1}H-NMR spectrum (400 MHz, $CDCl_3$) of **1**.

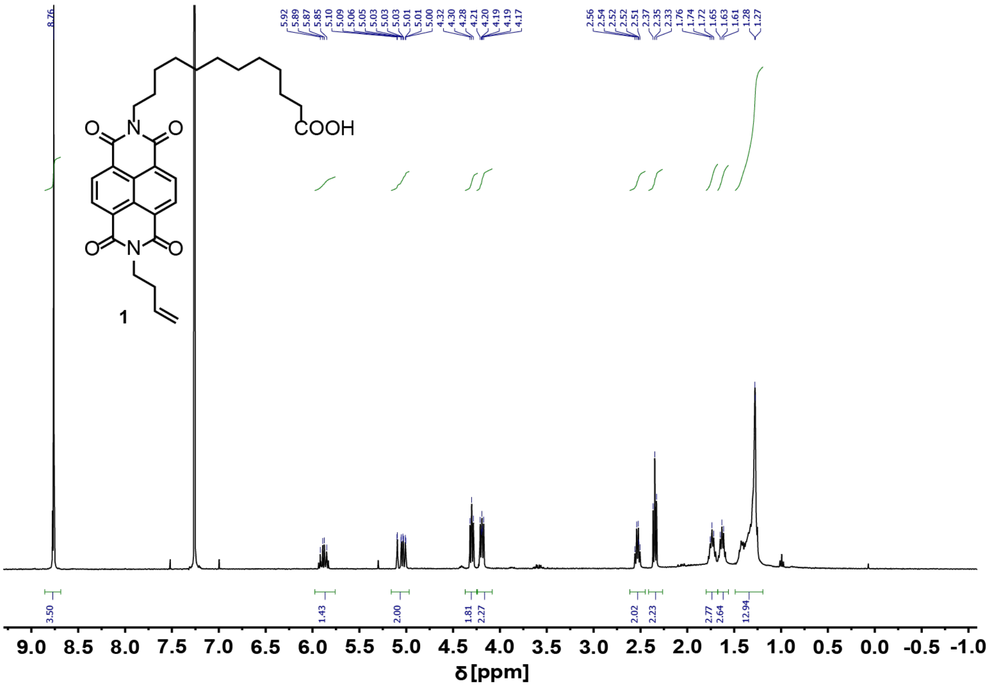

^{13}C-NMR spectrum (101 MHz, $CDCl_3$) of **1**.

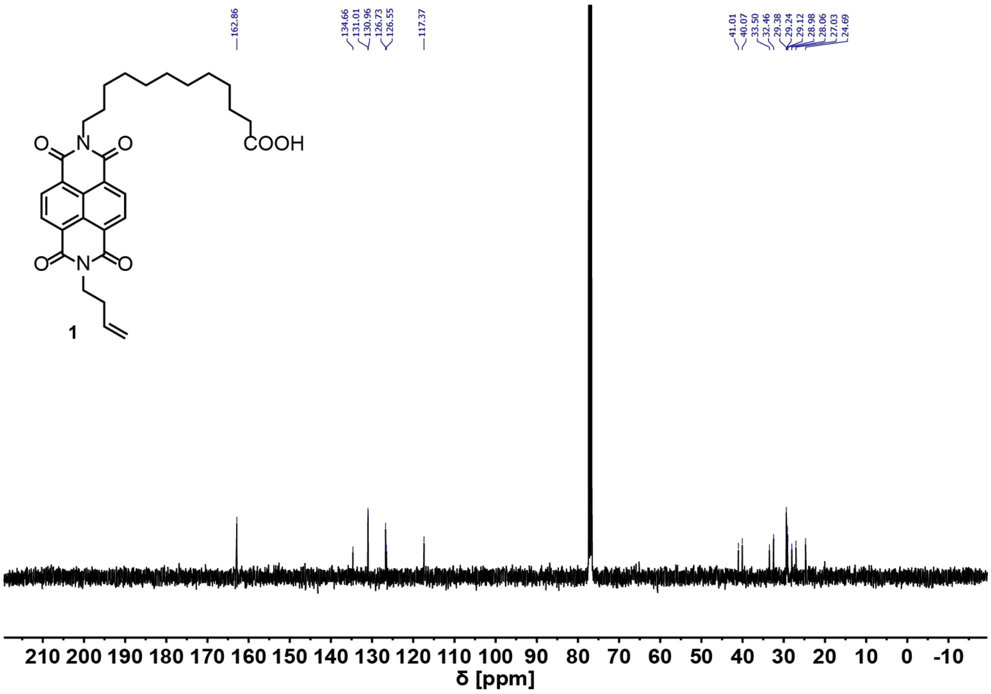

^{1}H-NMR spectrum (400 MHz, $CDCl_3$) of **2**.

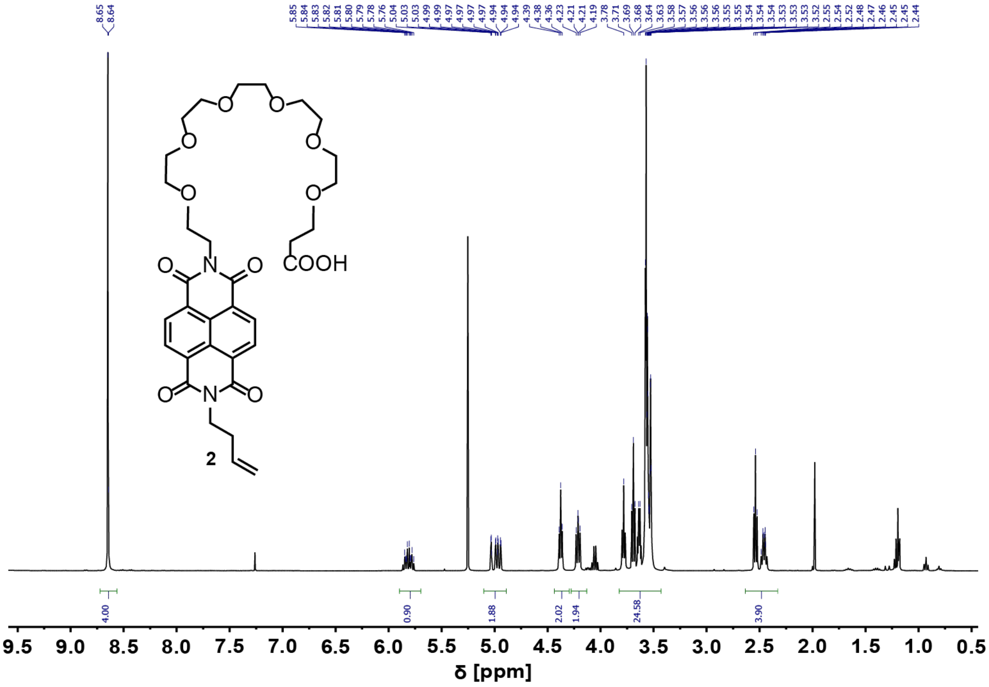

^{13}C-NMR spectrum (101 MHz, $CDCl_3$) of **2**.

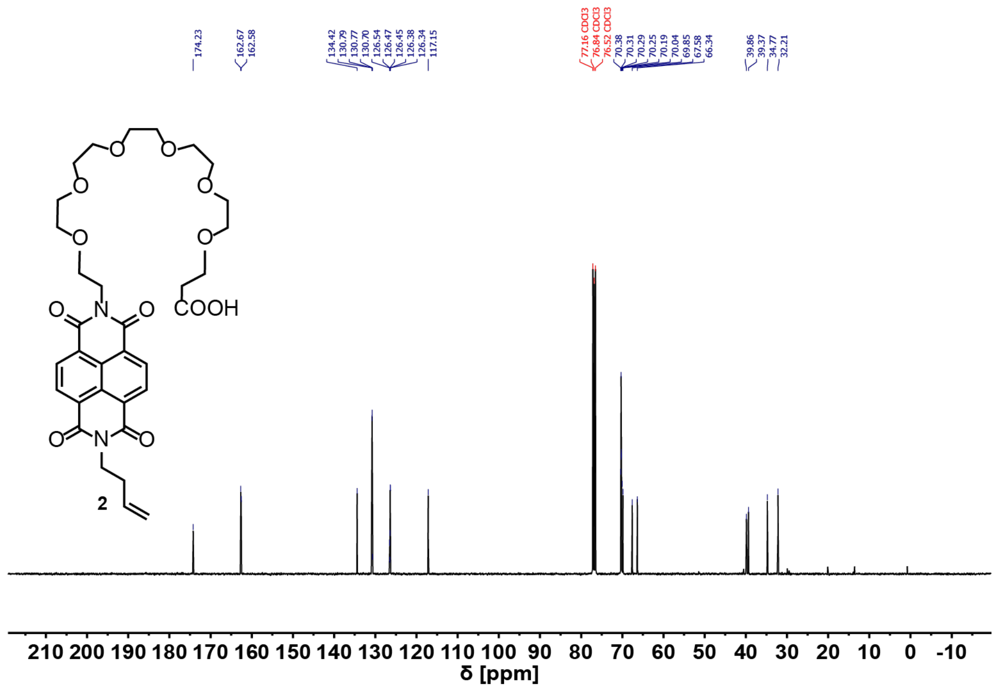

^{1}H-NMR spectrum (400 MHz, $CDCl_3$) of **3**.

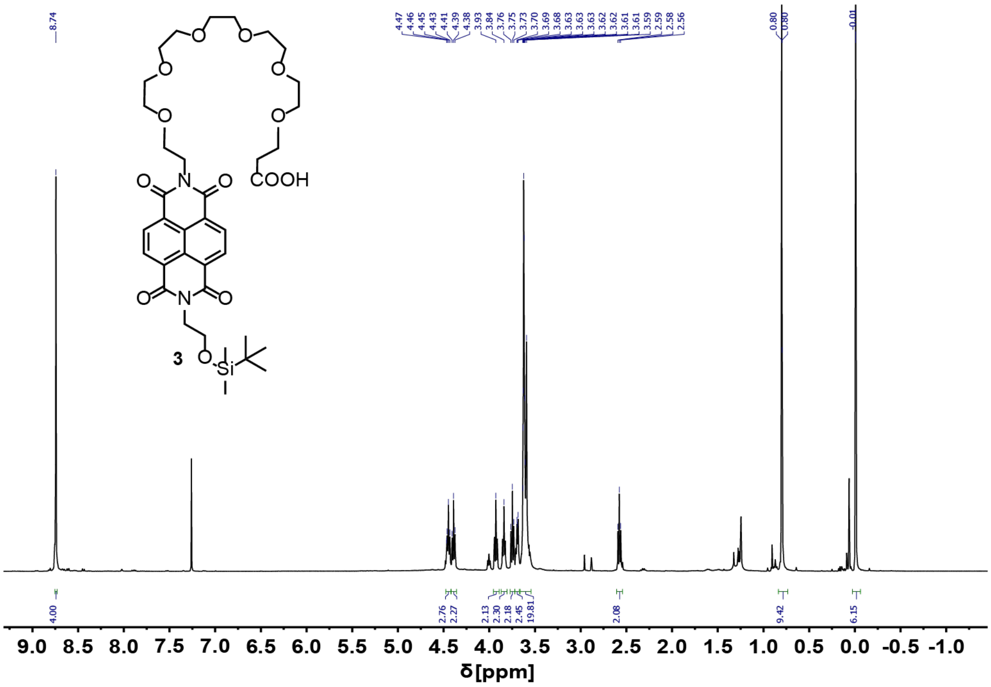

^{1}H-NMR spectrum (400 MHz, DMSO-d_6) of **4**.

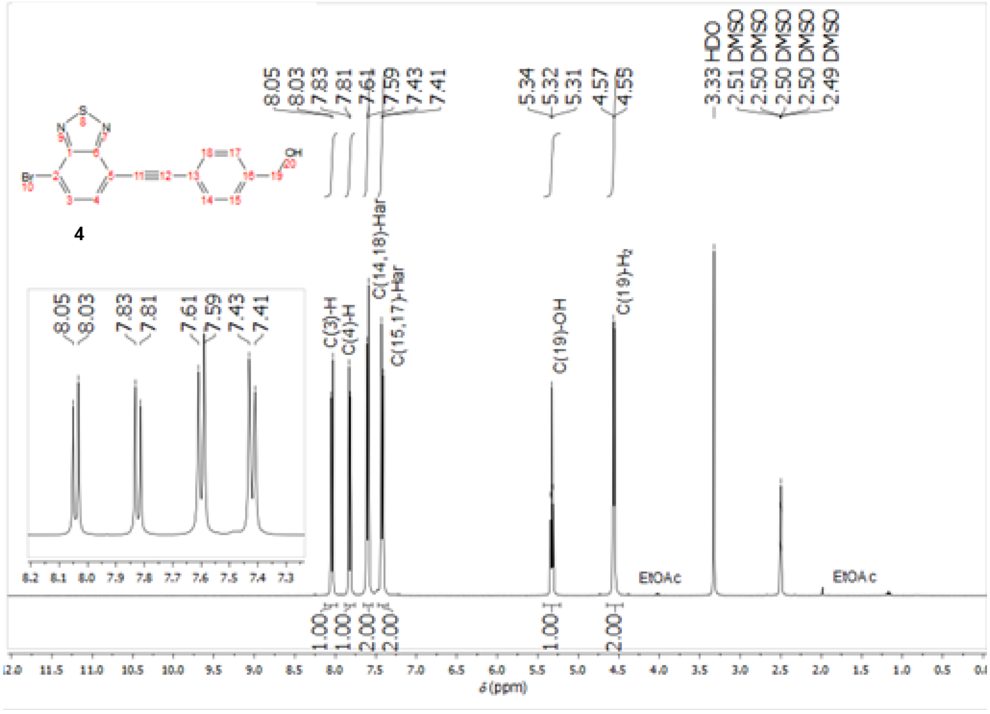

^{13}C-NMR spectrum (101 MHz, DMSO-d_6) of **4**.

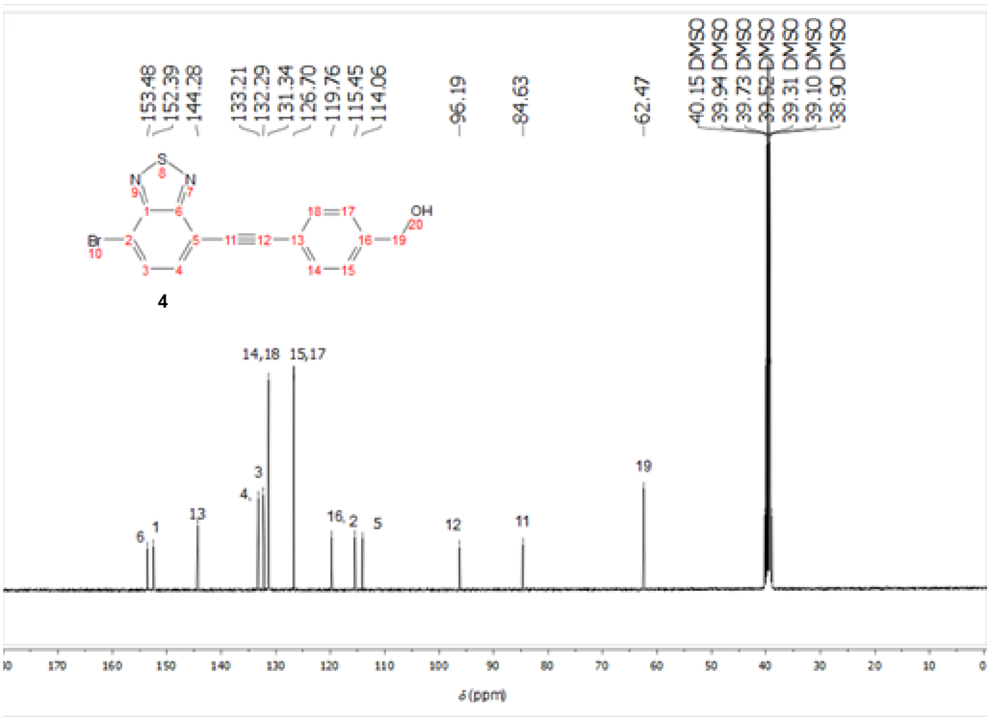

^{1}H-NMR spectrum (400 MHz, DMSO-d_6) of **5**.

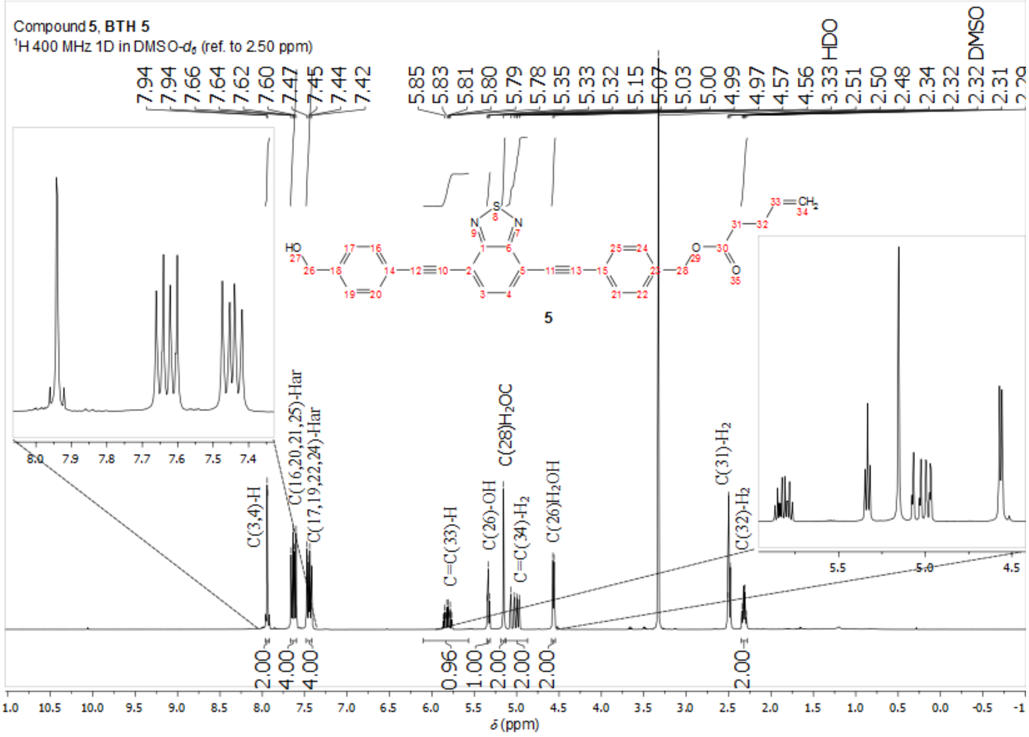

^{13}C-NMR spectrum (101 MHz, DMSO-d_6) of **5**.

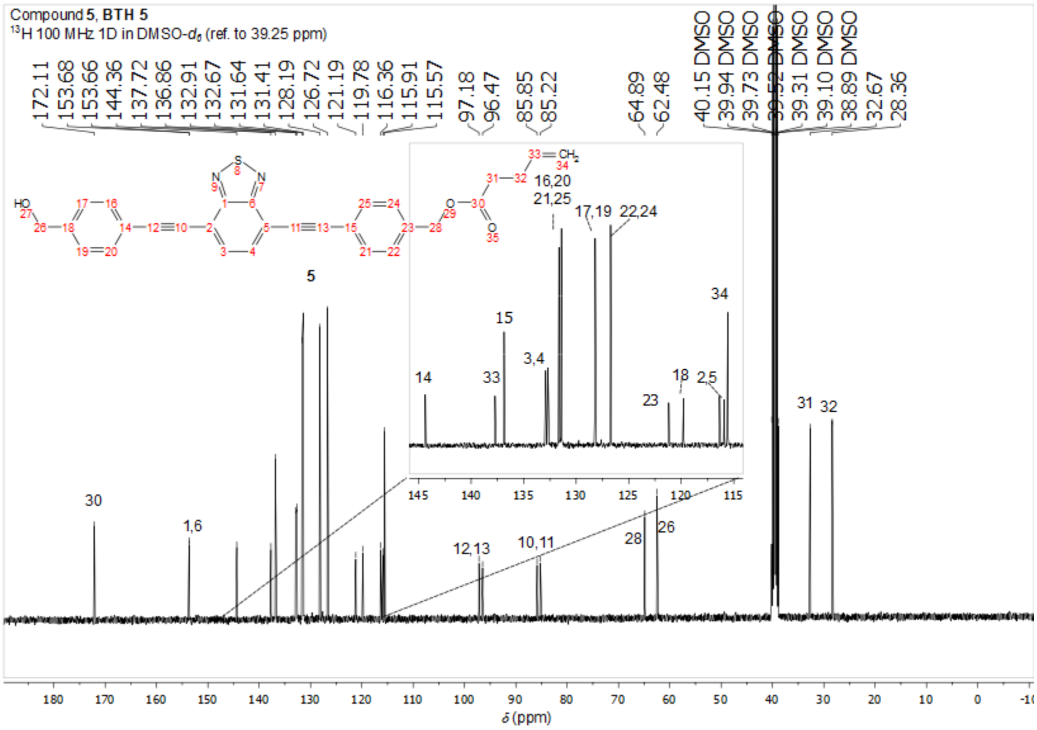

^{1}H-NMR spectrum (400 MHz, $CDCl_3$) of **6**.

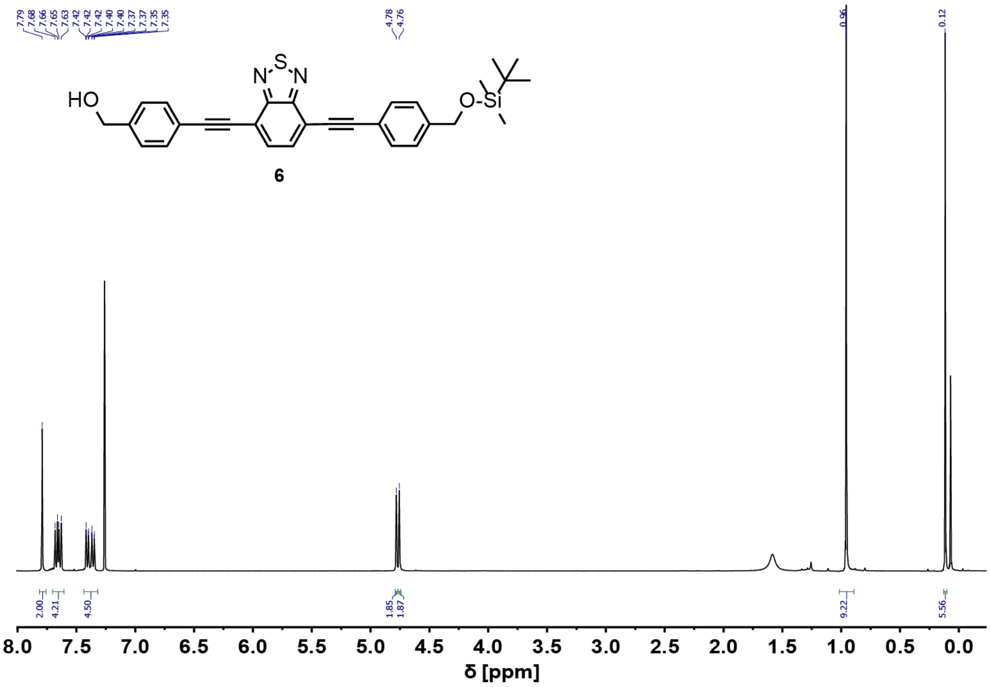

^{13}C-NMR spectrum (101 MHz, $CDCl_3$) of **6.**

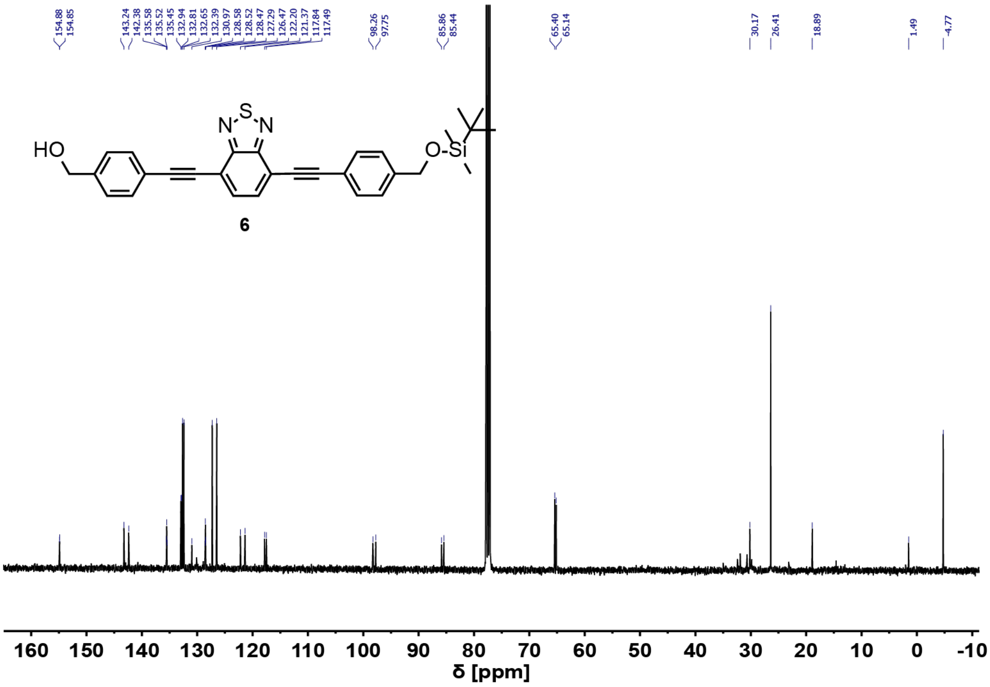

^{1}H-NMR spectrum (400 MHz, $CDCl_3$) of **7.**

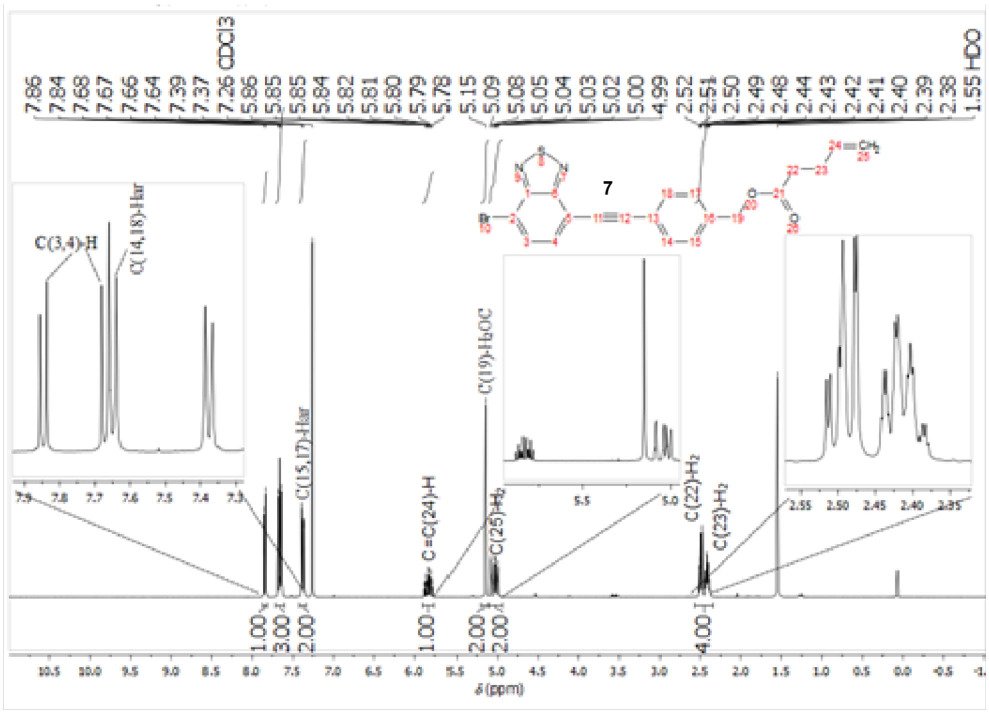

^{13}C-NMR spectrum (101 MHz, $CDCl_3$) of **7**.

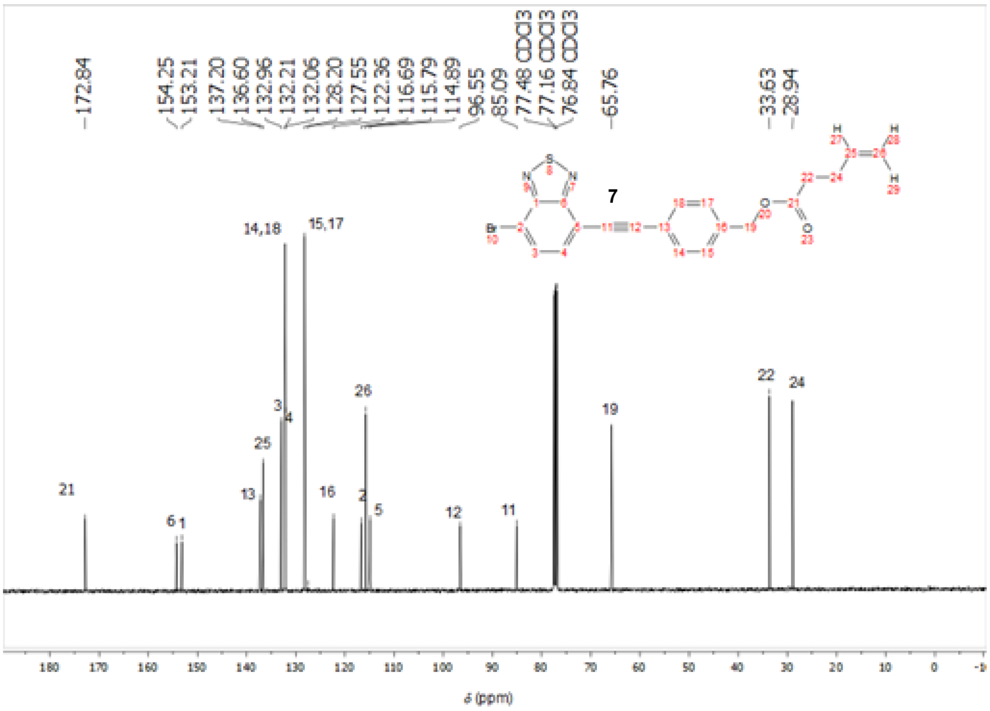

^{1}H-NMR spectrum (400 MHz, $CDCl_3$) of **8**.

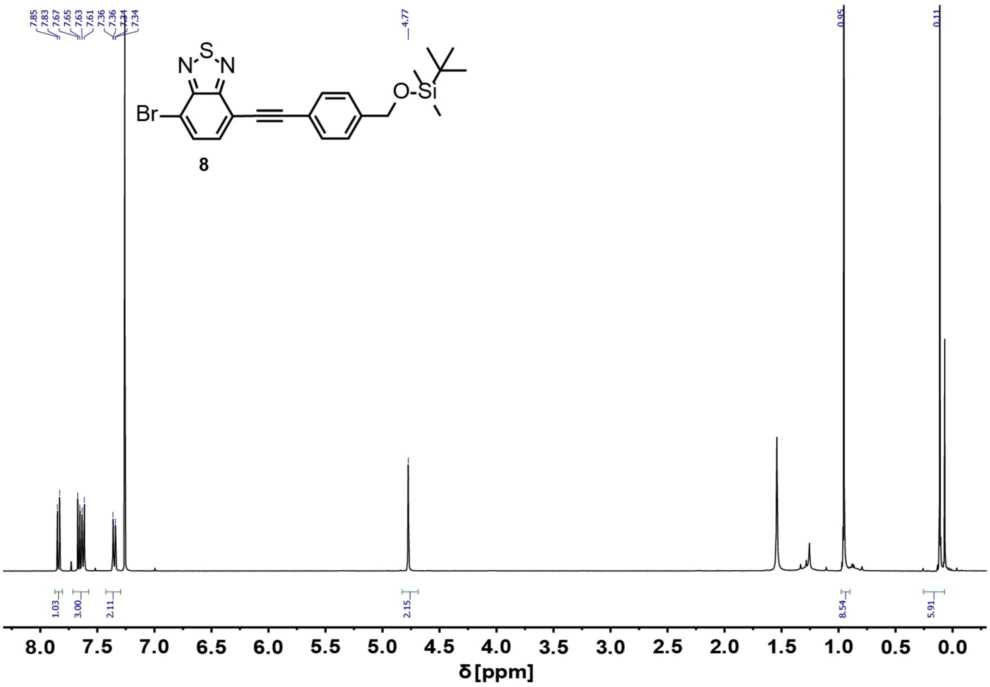

^{13}C-NMR spectrum (101 MHz, $CDCl_3$) of **8**.

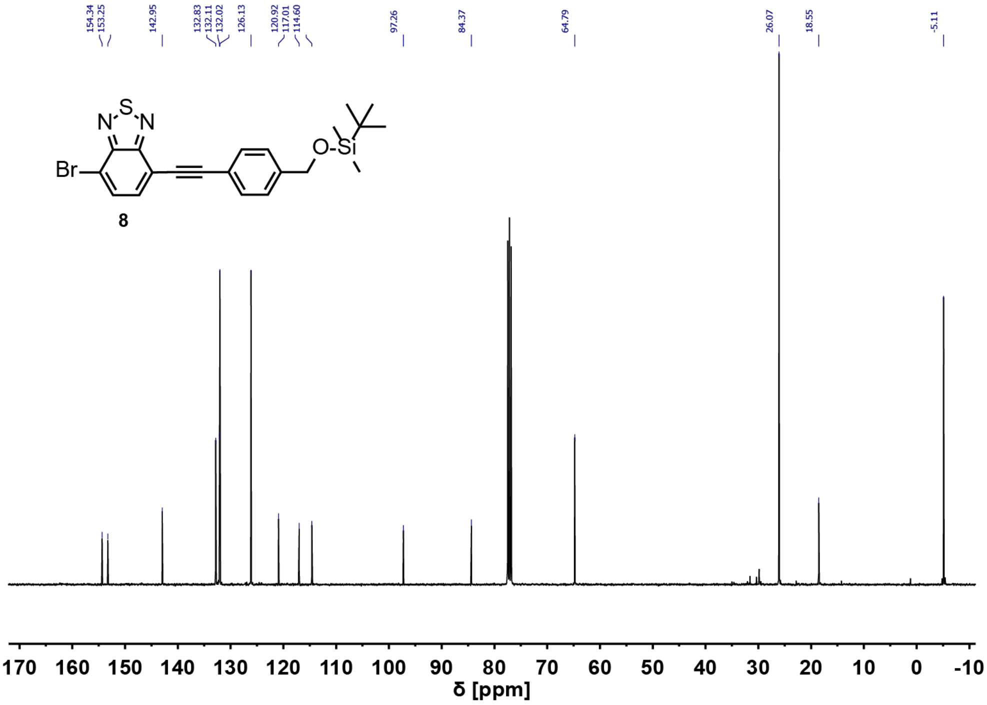

^{1}H-NMR spectrum (400 MHz, $CDCl_3$) of **9**.

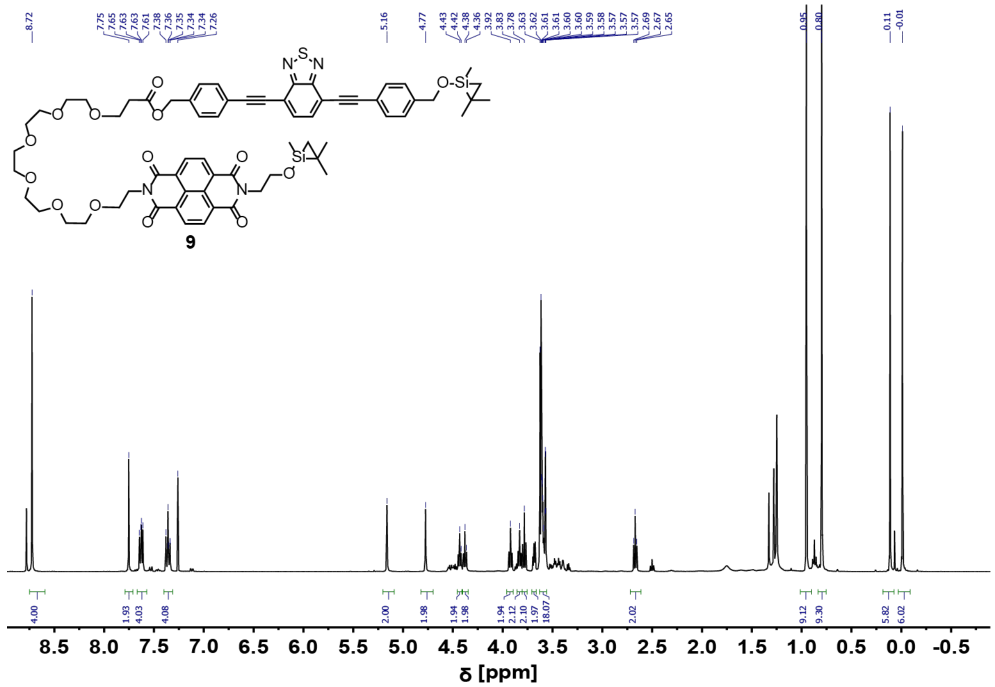

^{13}C-NMR spectrum (101 MHz, $CDCl_3$) of **9**.

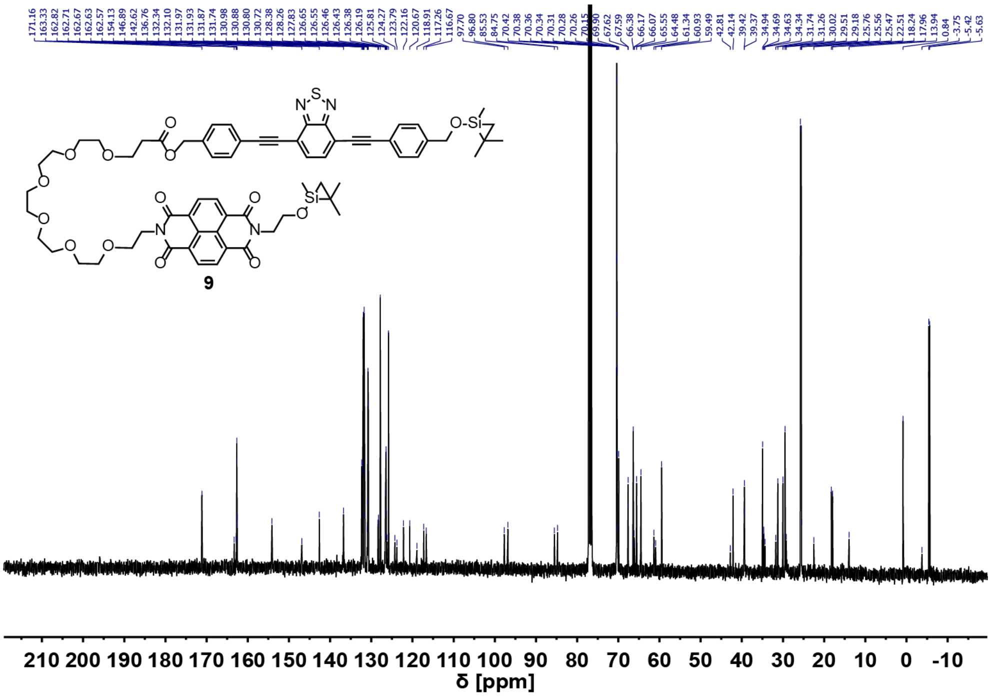

^{1}H-NMR spectrum (400 MHz, $CDCl_3$) of **D-C$_{12}$-A**.

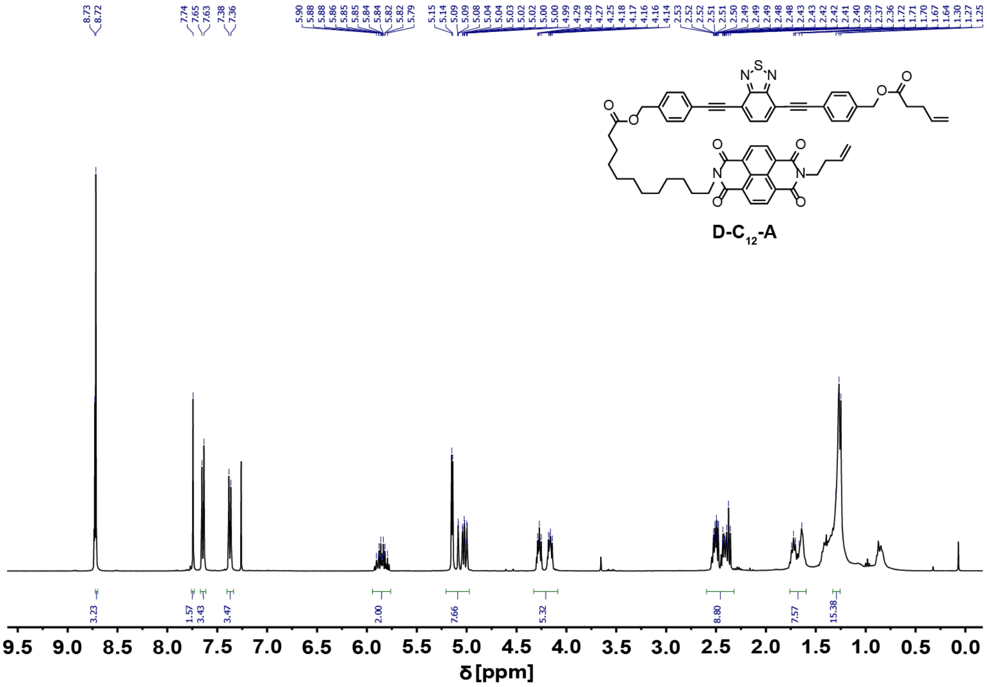

^{13}C-NMR spectrum (101 MHz, $CDCl_3$) of **D-C$_{12}$-A**.

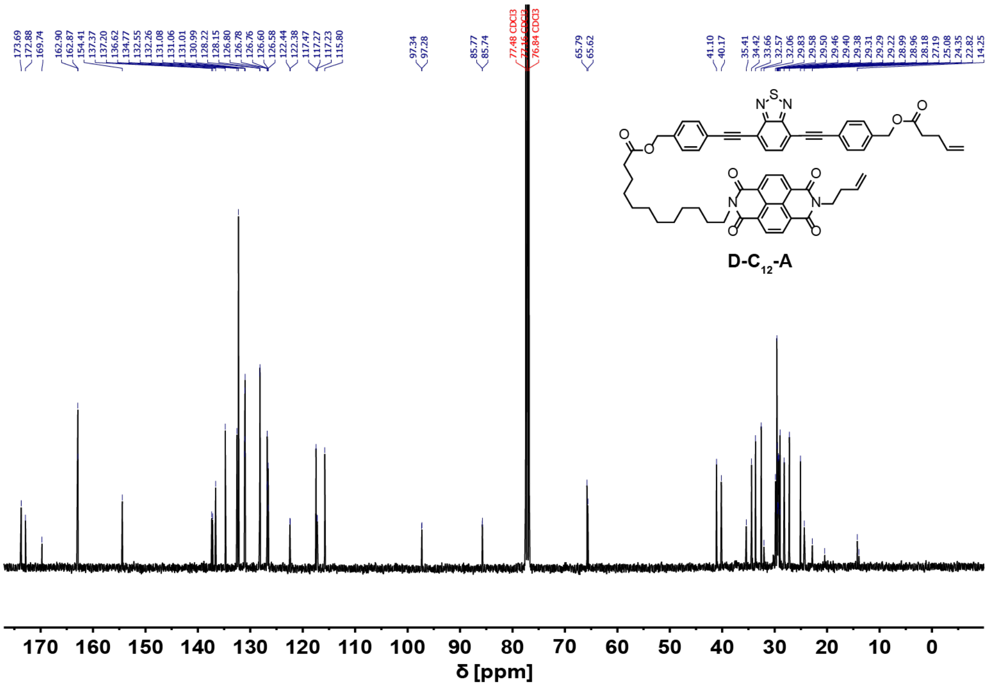

^{1}H-NMR spectrum (400 MHz, $CDCl_3$) of **D-EO$_6$-A**.

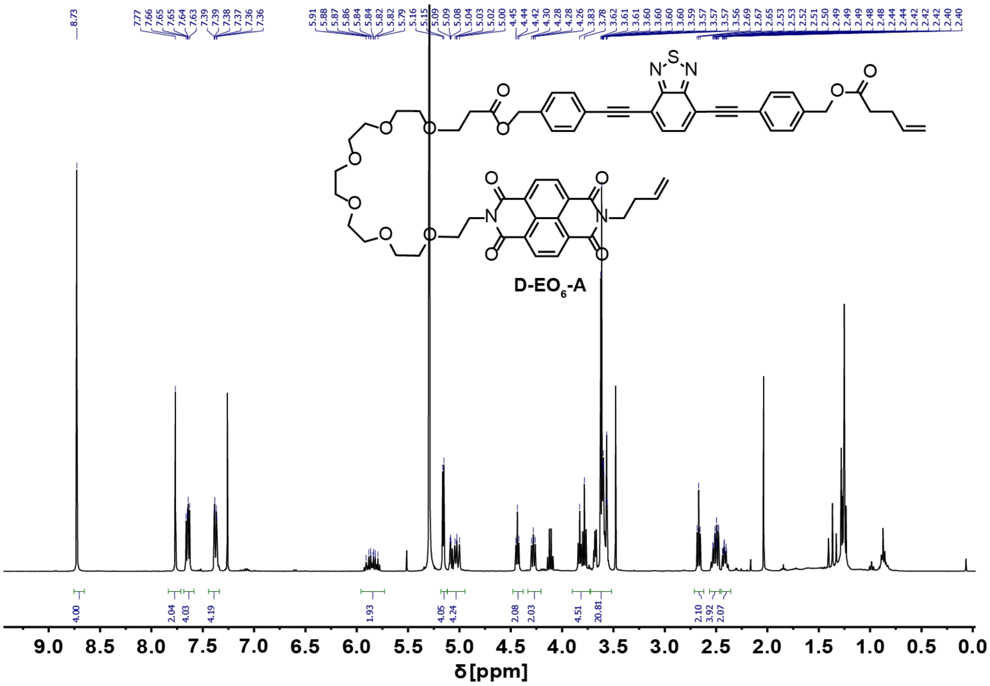

^{13}C-NMR spectrum (101 MHz, $CDCl_3$) of **D-EO$_6$-A**.

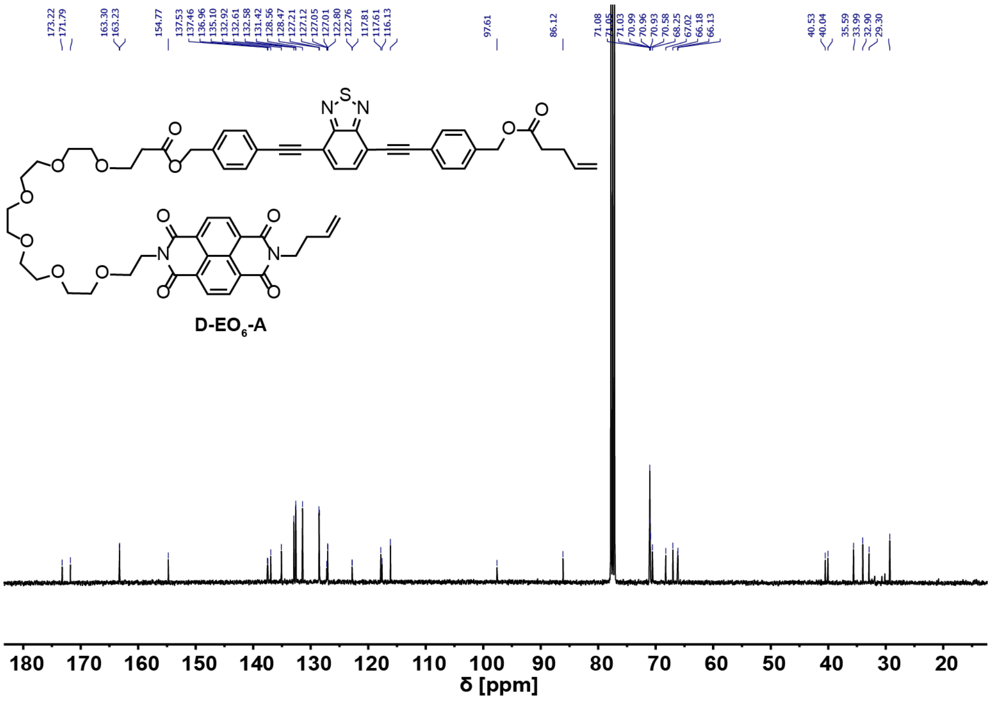

^{1}H-NMR spectrum (400 MHz, $CDCl_3$) of **HO-D-EO$_6$-A-OH**.

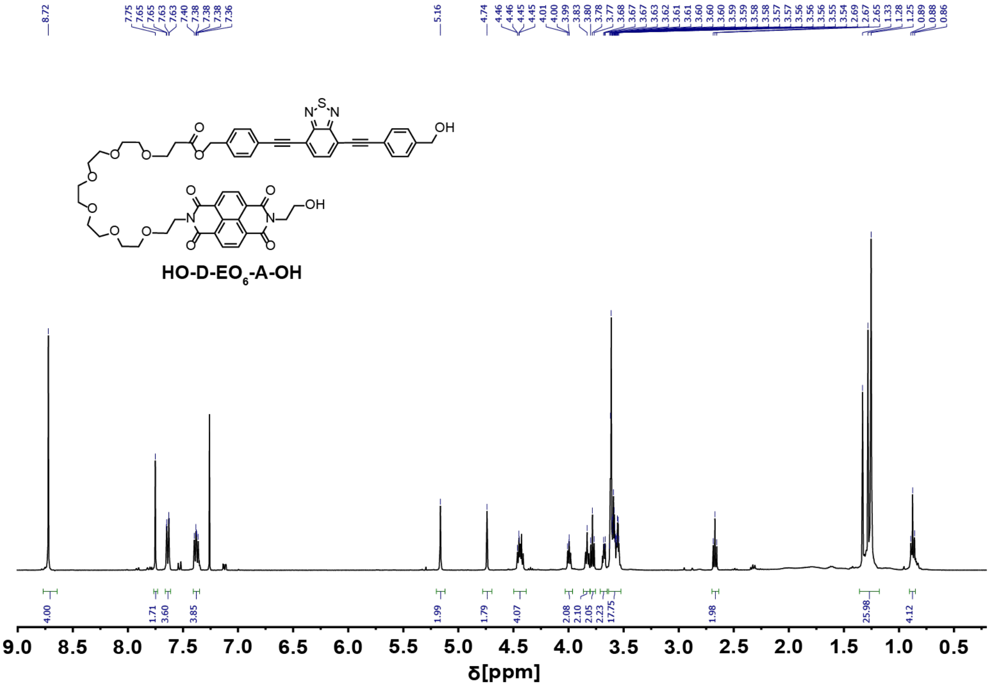

^{13}C-NMR spectrum (101 MHz, $CDCl_3$) of **HO-D-EO$_6$-A-OH**.

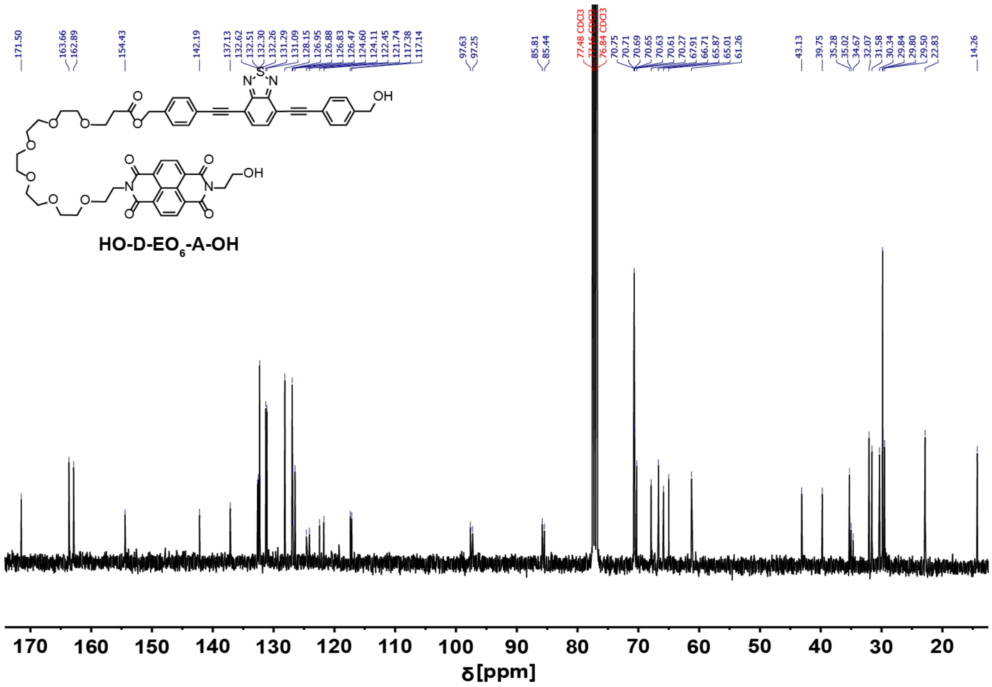

^{1}H-NMR spectrum (400 MHz, THF-*d*8) of **PU-D/A.**

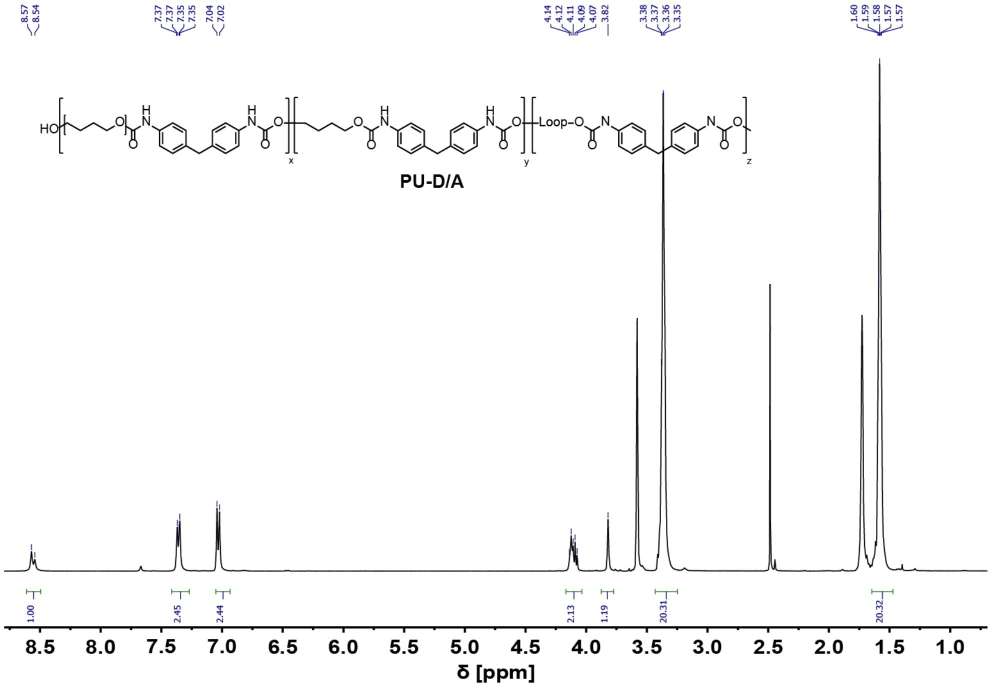

4. References

28. Tambara, K.; Ponnuswamy, N.; Hennrich, G.; Pantoş, G.D. Microwave-Assisted Synthesis of Naphthalenemonoimides and N-Desymmetrized Naphthalenediimides. *J. Org. Chem.* **2011**, *76*, 3338-3347.

36. Moore, J.S.; Stupp, S.I. Room temperature polyesterification. *Macromolecules* **1990**, *23*, 65-70.
